# Breaking down causes, consequences, and mediating effects of telomere length variation on human health

**DOI:** 10.1186/s13059-024-03269-9

**Published:** 2024-05-17

**Authors:** Samuel Moix, Marie C Sadler, Zoltán Kutalik, Chiara Auwerx

**Affiliations:** 1 Department of Computational Biology, UNIL, Lausanne, 1015 Switzerland; 2 Swiss Institute of Bioinformatics, Lausanne, 1015 Switzerland; 3 University Center for Primary Care and Public Health, Lausanne, 1015 Switzerland; 4 Center for Integrative Genetics, UNIL, Lausanne, 1015 Switzerland

**Keywords:** Telomeres, Mendelian randomization, UK Biobank, Complex traits, Lifespan, Aging, Lifestyle, Female reproduction, Mediation

## Abstract

**Background:**

Telomeres form repeated DNA sequences at the ends of chromosomes, which shorten with each cell division. Yet, factors modulating telomere attrition and the health consequences thereof are not fully understood. To address this, we leveraged data from 326,363 unrelated UK Biobank participants of European ancestry.

**Results:**

Using linear regression and bidirectional univariable and multivariable Mendelian randomization (MR), we elucidate the relationships between leukocyte telomere length (LTL) and 142 complex traits, including diseases, biomarkers, and lifestyle factors. We confirm that telomeres shorten with age and show a stronger decline in males than in females, with these factors contributing to the majority of the 5.4% of LTL variance explained by the phenome. MR reveals 23 traits modulating LTL. Smoking cessation and high educational attainment associate with longer LTL, while weekly alcohol intake, body mass index, urate levels, and female reproductive events, such as childbirth, associate with shorter LTL. We also identify 24 traits affected by LTL, with risk for cardiovascular, pulmonary, and some autoimmune diseases being increased by short LTL, while longer LTL increased risk for other autoimmune conditions and cancers. Through multivariable MR, we show that LTL may partially mediate the impact of educational attainment, body mass index, and female age at childbirth on proxied lifespan.

**Conclusions:**

Our study sheds light on the modulators, consequences, and the mediatory role of telomeres, portraying an intricate relationship between LTL, diseases, lifestyle, and socio-economic factors.

**Supplementary Information:**

The online version contains supplementary material available at 10.1186/s13059-024-03269-9.

## Background

Aging represents a leading risk factor for diseases such as cancer, cardiovascular diseases, and neurodegeneration [1]. Chronological age fails to account for individual differences in aging rates and vulnerability to diseases [2]. Biological age intends to address this limitation by reflecting the physiological state of an individual and accounting for variations in cellular and tissue health. Several biomarkers can be used to estimate biological age, with DNA methylation being particularly popular as it can be measured across different tissues, and is sensitive to both disease states and environmental factors [3–5]. However, given the complex nature of the aging process, additional biomarkers beyond DNA methylation are required to fully understand the underlying causes and mechanisms of aging and age-related diseases [6].

One such biomarker is telomere length. Telomeres are DNA repeats at chromosome ends that act as protective caps against genomic degradation. As organisms age, they undergo an increasing number of cell divisions, leading to an incremental decrease in telomere length. Acting as mitotic clocks, telomeres shorten until they reach a critical length, triggering cellular senescence and/or apoptosis [7]. Consequently, shorter telomeres have been associated with lifestyle factors, including smoking [8], reduced physical activity [9], high processed meat and low fruit consumption [10, 11], as well as a wide range of diseases, from pulmonary [12], renal [13], and metabolic [14] disorders to cancer [15, 16]. Paradoxically, longer telomeres have also been associated with poor health outcomes, especially cancers [17]. However, most studies so far were limited in the number of studied traits, relied on small sample sizes, and did not probe the directionality of the established associations.

Recently, efforts to assess leukocyte telomere length (LTL) in large population biobanks have allowed comprehensive exploration of its relationships with lifestyle factors and health outcomes. Performing an LTL phenome-wide association study in 62,271 participants from the biobank of Vanderbilt University Medical Center (BioVU) and Marshfield Clinic’s Personalized Medicine Research Project (PMRP), Allaire et al. identified associations with 67 phenotypes and showed that both shorter and longer telomeres associated with increased mortality [18]. Release of LTL measurements for $\sim$500,000 UK Biobank (UKBB) participants [19] and the companion first large-scale telomere length genome-wide association study (GWAS) [20] prompted investigation of the impact of LTL on hundreds of traits using phenome-wide Mendelian randomization (MR) [20, 21]. These studies showed that longer LTL increases risk for neoplastic and genitourinary diseases while shorter LTL increases risk for respiratory, digestive, and cardiovascular disorders [20, 21], with about 40% of these associations confirmed when using FinnGen disease association summary statistics [21].

**Fig. 1:**
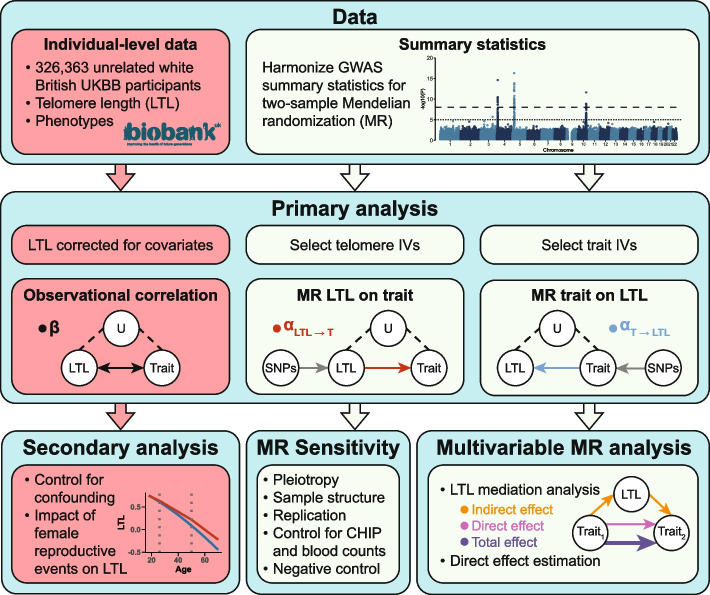
Schematic representation of the study’s workflow. Red and light green boxes denote steps using individual-level phenotypic data from the UK Biobank and genome-wide association studies (GWAS) summary statistics, respectively. Top: Data extraction process. Middle: Analyses focused on leukocyte telomere length (LTL) trait relationships including observational correlation ($\beta$; black), Mendelian randomization (MR) to assess the impact of LTL on traits ($\alpha _{LTL \rightarrow T}$; red), and MR to assess the impact of traits on LTL ($\alpha _{{T \rightarrow LTL}}$; blue). LTL covariates comprise age, age^2^, genotyping array, sex, and the interaction of the latter with the priors. U = unmeasured confounding factors; IVs = instrumental variables, i.e., genetic variants with genome-wide significant association to the considered trait. SNPs = single nucleotide polymorphisms. Bottom: Follow-up analyses include exploring the association of female reproductive events with LTL, sensitivity analyses (i.e., implementation of seven complementary MR methods, replication using independent LTL summary statistics [18], controlling for confounding by blood-related traits, and evaluating the MR effect of LTL on sex as a negative control), and perform mediation analysis through multivariable MR. Note that for mediation analyses, both exposure and mediator are instrumented. CHIP = clonal hematopoiesis of indeterminate potential

Our study builds on this body of work by dissecting observational correlations between LTL and 142 traits into causes and consequences through a robust bidirectional MR causal framework (Fig. 1). Additionally, we used multivariable Mendelian randomization (MVMR) to disentangle the interplay between LTL and various traits, with a particular focus on the mediating role of LTL in longevity. Together, we identify traits influencing LTL, and how in turn the latter impacts the human phenome, contributing to a deeper understanding of telomere biology and its relation to health.

## Results

### Age and sex are the main predictors of LTL variability

Consistent with previous research [22], LTL significantly associated with both age ($\widehat{\beta} = \text{-}0.023; p < 2.2e\text{-}308$) and sex ($\widehat{\beta} = 0.091; p < 2.2e\text{-}308$), with a stronger ($p_{\text {diff}} = 1.4e\text{-}25$) decline over time in males ($\widehat{\beta} _{males}= \text{-}0.025$) than in females ($\widehat{\beta} _{females}= \text{-}0.021$) (Additional file 1: Fig. S1). To further explore factors contributing to LTL variability, we included 80 traits (Additional file 2: Table S1) with < 7% missingness rates as predictor variables in a Lasso regression model. Traits retained included age, sex, educational attainment (EA), waist-to-hip ratio (WHR), insulin-like growth factor 1 (IGF-1), urate, and cystatin C levels, along with four blood parameters (Table 1; see the “Predictors of LTL variability” section). Among these, LTL was found to be positively associated with female sex, higher EA, and higher IGF-1 levels, while it negatively correlated with the remaining traits. Age and sex accounted for 4.33% of the observed variance in LTL. Incorporating the nine additional above-mentioned traits increased the explained variance to 5.39%. Repeating the analysis with missingness rate thresholds of 5% and 10% retained twelve and seven traits in addition to age and sex, which together explain 5.42% and 5.36% of variability in LTL, respectively, confirming the limited predictive power of the phenome over LTL variability (Additional file 2: Table S1).

**Table 1:** Major associations with LTL. Effect sizes with 95% confidence intervals (CI) from a joint linear regression model for traits with less than 7% missing data retained as significant LTL predictors by Lasso analysis. EA = educational attainment; IGF-1 = insulin-like growth factor 1; WHR = waist-to-hip ratio; MCH = mean corpuscular hemoglobin

Trait	Estimate	Lower 95% CI	Upper 95% CI	*P*-value
Female	0.071	0.066	0.077	$2.33 \times 10^{\text{-}160}$
EA	0.042	0.038	0.046	$1.95 \times 10^{\text{-}111}$
IGF-1	0.028	0.024	0.032	$3.57 \times 10^{\text{-}49}$
WHR	$\text{-}0.002$	$\text{-}0.007$	0.004	$5.43 \times 10^{\text{-}1}$
Urate	$\text{-}0.009$	$\text{-}0.014$	$\text{-}0.004$	$1.16 \times 10^{\text{-}4}$
Monocyte count	$\text{-}0.017$	$\text{-}0.021$	$\text{-}0.013$	$4.89 \times 10^{\text{-}17}$
Eosinophil count	$\text{-}0.021$	$\text{-}0.025$	$\text{-}0.017$	$1.00 \times 10^{\text{-}28}$
Cystatin C	$\text{-}0.026$	$\text{-}0.030$	$\text{-}0.022$	$4.93 \times 10^{\text{-}33}$
Lymphocyte count	$\text{-}0.039$	$\text{-}0.043$	$\text{-}0.035$	$7.43 \times 10^{\text{-}87}$
MCH	$\text{-}0.058$	$\text{-}0.062$	$\text{-}0.055$	$4.98 \times 10^{\text{-}223}$
Age	$\text{-}0.155$	$\text{-}0.159$	$\text{-}0.151$	$< 2.2 \times 10^{\text{-}308}$

### LTL broadly associates with complex traits

**Fig. 2:**
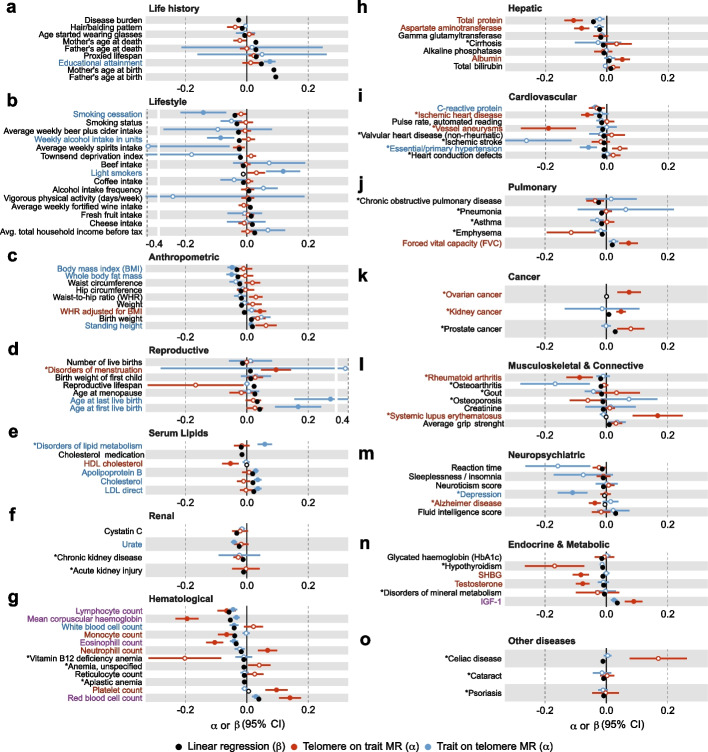
Observational and causal associations between traits and LTL. Estimates (*x*-axis) with 95% confidence intervals (CI) for traits (*y*-axis) with at least one strictly significant ($p < 0.05/141 = 3.5e\text{-}4$) association with LTL across the observational correlation (linear regression; $\beta$; black) and inverse-variance weighted (IVW) Mendelian randomization (MR) estimates of LTL on trait ($\alpha$; red) and trait on LTL ($\alpha$; blue) are shown. Strictly significant effects are shown as full circles; otherwise as empty circles. Traits are colored according to their MR effects, with red, blue, or purple indicating a significant LTL to trait, trait to LTL, or bidirectional effect. For diseases (*), one standard deviation ($\text {SD}$) change in LTL corresponds to one $\log (OR)$ change, implying a scale of $\text {SD}_{\text {LTL}} / \log (OR)$ for the effects of diseases on LTL, and $\log (OR) / \text {SD}_{\text {LTL}}$ for the effect of LTL on the disease, so that observational effects and MR effects are not directly comparable (Additional file 2: Table S2)

Considering the strong correlation between age and sex with LTL, we adjusted LTL for age, age^2^, genotyping array, sex, and the interaction of the latter with the priors. We then regressed adjusted LTL (hereafter simply referred to as LTL) on 166 traits through linear regression, identifying 100 significant associations ($p < 0.05/141 = 3.5e\text{-}4$; Additional file 2: Table S2). We observed a negative association between the disease burden and LTL ($\widehat{\beta} = \text{-}0.027; p = 1.2e\text{-}52$), suggesting that LTL acts as a global health indicator. The largest effect sizes were noted for father’s ($\widehat{\beta} = 0.094; p = 4.4e\text{-}144$) and mother’s ($\widehat{\beta} = 0.088; p = 8.5e\text{-}216$) age at birth, which positively associated with LTL (Fig. 2a). To test confounding by socio-economic status (SES), we jointly modeled LTL as a function of both parental ages at birth, participant’s age, sex, and age-sex interaction, and EA. We found that the associations with parental ages at birth were independent of the participant’s education level (Additional file 1: Fig. S2), which likely echoes parental EA [23] and indirectly affects parental age at birth. This suggests that the association is likely not confounded by SES and is genuinely driven by older parental age at birth. As these traits cannot be genetically instrumented, MR is not applicable. As such, the observational nature of our analysis prevents us from further dissecting the effects of paternal versus maternal age at birth on LTL. Next, for the 142 traits with available GWAS summary statistics and at least two instrumental variables (IVs), we inferred bidirectional causal relationships through univariable MR, identifying 23 significant causal effects of traits on LTL ($\widehat{\alpha} _{T \rightarrow LTL}$) and 24 significant effects of LTL on traits ($\widehat{\alpha} _{LTL \rightarrow T}$) ($p < 0.05/141 = 3.5e\text{-}4$; Fig. 2 and Additional file 2: Table S2).

### Sensitivity analyses

To ensure the robustness and reliability of our results, we gauged the reliability of inverse-variance weighted (IVW) significant associations through several approaches (Fig. 1). First, we estimated robustness towards MR assumption violation. We applied four additional MR methods implemented in the TwoSampleMR package (MR Egger, simple mode, weighted median, and weighted mode), as well as MR-PRESSO (Additional file 1: Figs. S3–4). To mitigate pleiotropy bias, we further implemented a custom approach based on Steiger filtering that requires IVs to have a stronger association with the exposure (i.e., LTL) than with any of the other 152 traits analyzed through MR (see the “Methods” section and Additional file 1: Fig. S5). To determine the impact of sample structure, and more notably sample overlap, we ran MR-APSS (which also accounts for pleiotropy) and replicated IVW results using independent LTL summary statistics from BioVU/PMRP [18]. Estimates were globally consistent across all methods (Additional file 1: Fig. S3–4). Regarding replication in BioVU/PMRP, the smaller sample size ($N = 62, 271$) resulted in larger confidence intervals (CI), yet the correlation between effect sizes remained high ($\rho _{LTL \rightarrow T} = 0.930; \rho _{T \rightarrow LTL} = 0.874$). Specifically, 9 LTL on trait and 5 trait on LTL effects strictly replicated ($p < 0.05/141 = 3.5e\text{-}4$), while 17 and 12 reached nominal significance, respectively. Only the effect of white blood cell (WBC) counts on LTL had a significantly different effect size ($p_{\text {diff}} < 0.05/47 = 0.001$).

Second, we sought to assess if our results could be confounded by hematological factors, given that we use telomere length assessed in leukocytes. We therefore adjusted LTL for eosinophil, lymphocyte, monocyte, neutrophil, platelet, red blood cell, reticulocyte, and WBC counts in addition to core covariates. Regressing this new variable on the same 158 traits (i.e., 166 traits, excluding the 8 blood count traits we corrected for), we obtained highly similar effect sizes ($\rho = 0.98$). Only associations with smoking status ($p_{\text {diff}} = 1.4e\text{-}9$), smoking cessation ($p_{\text {diff}} = 5.7e\text{-}6$), as well as mean corpuscular hemoglobin (MCH; $p_{\text {diff}} = 3.3e\text{-}26$) were significantly reduced ($p_{\text {diff}} < 0.05/141 = 3.5e\text{-}4$), yet remained significant. Association with total bilirubin ($p_{\text {diff}} = 1.4e\text{-}5$) was lost, while the one with phosphate levels ($p_{\text {diff}} = 8.0e\text{-}5$) became significant (Additional file 1: Fig. S6). As a relationship between LTL and clonal hematopoiesis of indeterminate potential (CHIP) has been suggested [24, 25], we conducted bidirectional MR analysis to assess whether CHIP [26] could confound LTL associations. While long LTL had a causal impact on CHIP incidence ($\widehat{\alpha} _{LTL \rightarrow T} = 0.147; p = 3.0e\text{-}9$), we did not identify a reverse effect ($\widehat{\alpha} _{T \rightarrow LTL} = 0.040; p = 0.618$). This suggests that confounding of our MR analyses by CHIP is an unlikely scenario. MVMR analysis with blood counts (with significant association with LTL), MCH, CHIP, and LTL as exposures against LTL-impacted traits also did not reveal significant effect changes in effect sizes, confirming that neither CHIP nor other hematological parameters biased our results (Additional file 2: Table S3).

Third, we performed a negative control. As sex of an individual is determined prior to adult LTL, we should not observe any causal link from LTL to sex [27]. As expected, we did not find a significant causal IVW MR effect of LTL on sex ($p = 0.656$). To conclude, the broad range of sensitivity analyses we performed showed that our results are globally robust to assumption violation and confounding, allowing their biological interpretation.

### Modulators of LTL

#### Lifestyle and environmental factors

Our results are overall concordant with deleterious lifestyle habits leading to shorter LTL (Fig. 2b). A negative correlation was observed between smoking cessation and LTL ($\widehat{\beta} = \text{-}0.039 ; p = 9.4e\text{-}50$), mirrored by a detrimental causal effect of failure to quit smoking on LTL ($\widehat{\alpha} _{T \rightarrow LTL} = \text{-}0.142; p = 1.8e\text{-}4$). Alcohol consumption, measured as total weekly intake of alcohol units, also exhibited a negative causal effect on LTL ($\widehat{\alpha} _{T \rightarrow LTL} = \text{-}0.086; p = 1.3e\text{-}4$), while beef consumption showed a mere associative ($\widehat{\beta} = \text{-}0.012; p = 2.4e\text{-}11$) but no causal link ($p = 0.223$). Conversely, healthy habits such as high fresh fruit intake ($\widehat{\beta} = 0.014; p = 6.4e\text{-}15$) and physical activity ($\widehat{\beta} = 0.007; p = 1.7e\text{-}4$) displayed positive associations with LTL, as did SES captured by average household income ($\widehat{\beta} = 0.025; p = 1.1e\text{-}40$) or EA ($\widehat{\beta} = 0.047; p = 1.9e\text{-}155$), even though only the latter showed clear causal evidence ($\widehat{\alpha} _{T \rightarrow LTL} = 0.075; p = 2.2e\text{-}15$). Our data also suggest that the psychological state of an individual can impact LTL as depression causes shorter LTL ($\widehat{\alpha} _{T \rightarrow LTL} = \text{-}0.110; p = 8.0e\text{-}6$). We speculate that depression could accelerate LTL shortening by influencing lifestyle factors that promote oxidative stress and inflammation, both critical modulators of LTL [7, 28, 29]. This hypothesis is supported by a negative causal effect of the inflammation marker C-reactive protein (CRP) on LTL ($\widehat{\alpha} _{T \rightarrow LTL} = \text{-}0.037; p = 9.3e\text{-}10$). While it is challenging to genetically instrument lifestyle factors, we conducted mediation analyses to explore the extent to which smoking cessation, frequency of alcohol intake, body mass index (BMI), and CRP levels mediate the effect of depression on LTL. We found that only CRP significantly mediated part of the relationship between depression and LTL ($P_\text{M}=14.5\%;95\%\;\text{CI = }\lbrack3.3\%;32.1\%\rbrack$). Overall, these results highlight the significant impact of lifestyle and environmental factors on LTL and support the paradigm that exposures typically considered as deleterious lead to shorter LTL.

#### Anthropometric traits

We detect several associations with anthropometric traits (Fig. 2c). Body metrics such as BMI and body fat mass (BFM) demonstrated significant negative observational correlation (BMI: $\widehat{\beta} = \text{-}0.032; p = 2.4e\text{-}75$; BFM: $\widehat{\beta} = \text{-}0.029; p = 1.2e\text{-}60$) and causal effects on LTL (BMI: $\widehat{\alpha} _{T \rightarrow LTL} = \text{-}0.048; p = 4.9e\text{-}10$; BFM: $\widehat{\alpha} _{T \rightarrow LTL} = \text{-}0.050; p = 7.6e\text{-}9$). Conversely, a positive correlation was observed between LTL and height ($\widehat{\beta} = 0.018; p = 2.2e\text{-}24$), with MR analysis revealing a nominally significant effect of LTL on height ($\widehat{\alpha} _{LTL \rightarrow T} = 0.062; p = 4.5e\text{-}4$) and strictly significant effect of height on LTL ($\widehat{\alpha} _{T \rightarrow LTL} = 0.013; p = 4.0e\text{-}5$).

#### Female reproductive traits

**Fig. 3:**
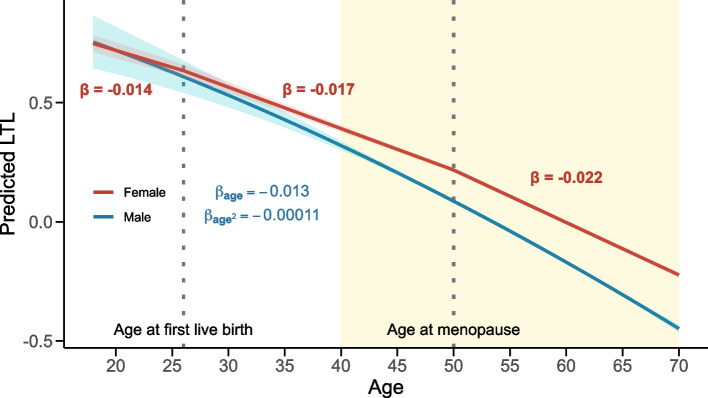
Schematic representation of LTL shortening across different female reproductive life phases. Relation ($\beta$) between predicted (i.e., regression model fit) standardized LTL (*y*-axis) and age (*x*-axis) across three female reproductive life periods (red). Dotted vertical lines indicate mean age at first live birth (26 years) and mean age at menopause (50 years). As a comparison, we depict the quadratic LTL regression in males ($\beta _{age}$; $\beta _{age^2}$; blue). 95% confidence intervals are shown for the predictions. Yellow background indicates the age range for which data are available (40–70 years) and used to build predictions; regions outside this range are extrapolated for males and estimated from age at first live birth and age at menopause information for females. The x-axis was set to begin at age 18, reflecting the shift in the rate of LTL decline after puberty [30]. This change cannot be accurately captured by our linear models, which are based on LTL measurements at older ages

Observational correlation between LTL and female reproductive traits including age at first (AFB; $\widehat{\beta} = 0.042; p = 1.4e\text{-}54$) and last (ALB; $\widehat{\beta} = 0.034; p = 2.5e\text{-}36$) live birth, reproductive lifespan ($\widehat{\beta} = 0.023; p = 3.7e\text{-}13$), age at menopause ($\widehat{\beta} = 0.026; p = 1.5e\text{-}16$), and menstrual disorders ($\widehat{\beta} = 0.011; p = 1.7e\text{-}5$) were observed (Fig. 2d). Testing for age at menarche showed no association with LTL ($\widehat{\beta} = 0.004; p = 0.113$). Only the effect of AFB ($p_{\text {diff}} = 6.7e\text{-}5$) and ALB ($p_{\text {diff}} = 0.001$) were significantly ($p_{\text {diff}} < 0.05/11 = 4.5e\text{-}3$) reduced after accounting for SES, even though they remained significant (Additional file 1: Fig. S7a). Both traits also causally influenced LTL (AFB: $\widehat{\alpha} _{T \rightarrow LTL} = 0.167; p = 1.2e\text{-}5$; ALB: $\widehat{\alpha} _{T \rightarrow LTL} = 0.272; p = 6.1e\text{-}6$), suggesting that timing of female reproductive events could modulate LTL. While these effects showed nominally significant MR-Egger intercepts, potentially indicating directional pleiotropy, the latter did not survive multiple testing correction (Additional file 2: Table S2). To explore this further, we compared LTL in women with and without children, finding shorter LTL in women who had given birth (Welch two-sample t-test: $p = 7.4e\text{-}11$). This suggests that childbirth could accelerate LTL shortening. We next divided female participants’ age into three reproductive periods: (1) premenopausal before first live birth, (2) premenopausal after first live birth, and (3) postmenopausal, and used the number of years spent in each period as predictors of LTL. LTL shortening accelerated over the course of these periods, with the weakest effect on LTL found for premenopausal years before childbirth ($\widehat{\beta} = \text{-}0.014; p = 3.6e\text{-}120$), followed by premenopausal years after childbirth ($\widehat{\beta} = \text{-}0.017; p = 7.1e\text{-}233$), and postmenopausal years ($\widehat{\beta} = \text{-}0.022; p < 2.2e\text{-}308$) (Fig. 3), in line with the hypothesis that female reproductive events trigger acceleration in LTL shortening.

#### Serum lipids

We found predominantly positive associations between LTL and serum lipid levels, i.e., apolipoprotein B (ApoB; $\widehat{\beta} = 0.019; p = 9.4e\text{-}25$), total cholesterol ($\widehat{\beta} = 0.019; p = 2.9e\text{-}27$), and low-density lipoprotein (LDL)-cholesterol ($\widehat{\beta} = 0.022; p = 4.2e\text{-}35$) (Fig. 2e). After adjusting for cholesterol-lowering drug use, the positive relation between LTL and both total and LDL-cholesterol decreased but remained significant (Additional file 1: Fig. S7b). ApoB ($\widehat{\alpha} _{T \rightarrow LTL} = 0.029; p = 2.6e\text{-}10$), total cholesterol ($\widehat{\alpha} _{T \rightarrow LTL} = 0.035; p = 1.5e\text{-}08$), and LDL-cholesterol ($\widehat{\alpha} _{T \rightarrow LTL} = 0.036; p = 4.7e\text{-}10$) levels also causally influenced LTL. Consistently, our findings suggest that disorders of lipid metabolism contribute to longer LTL ($\widehat{\alpha} _{T \rightarrow LTL} = 0.058; p = 5.8e\text{-}7$), reiterating the association between increased LTL and high serum lipid levels. Due to their correlated nature, MVMR including levels of LDL-cholesterol, ApoB, and triglycerides as exposures could not disentangle their individual contribution to LTL (Additional file 1: Fig. S8).

#### Urate

Urate levels, also retained as a relevant predictor of LTL by the Lasso regression analysis, displayed a negative association with LTL ($\widehat{\beta} = \text{-}0.025; p = 2.1e\text{-}44$). As previously reported [31], MR analyses showed that elevated urate levels decreased LTL ($\widehat{\alpha} _{T \rightarrow LTL} = \text{-}0.042; p = 9.4e\text{-}18$), possibly due to increased cellular stress and reactive oxygen species production [32] (Fig. 2f). The urate-LTL association was significantly mediated by CRP, confirming the role of inflammation in this process ($P_\text{M}=34.7\%;95\%\;\text{CI = }\lbrack17.1\%;55.6\%\rbrack$; Additional file 2: Table S4).

### Consequences of altered LTL

#### Blood cell counts

Hematological traits (e.g., WBC count: $\widehat{\beta} = \text{-}0.042; p = 2.9e\text{-}120$; and MCH: $\widehat{\beta} = \text{-}0.054; p = 1.7e\text{-}200$) are among the ones showcasing the strongest observational correlation with LTL (Fig. 2g). For four out of eleven significantly correlated blood traits, we identified bidirectional causal relationships with LTL, with less pronounced effects from traits on LTL (e.g., MCH: $\widehat{\alpha} _{LTL \rightarrow T} = \text{-}0.195; p = 2.2e\text{-}24$; $\widehat{\alpha} _{T \rightarrow LTL} = \text{-}0.034; p = 5.2e\text{-}10$). While effects of LTL on MCH, eosinophil, platelet, and red blood cell counts were robust across multiple MR methods (Additional file 1: Fig. S3), the effects of blood traits on LTL did not necessarily pass Bonferroni correction in all sensitivity analyses (Additional file 1: Fig. S4). Given the previously described analyses (see the “Sensitivity analyses” section), it appears that these blood traits do not confound the other observed relationships with LTL.

#### Hepatic biomarkers

LTL associated with levels of the hepatic biomarkers aspartate aminotransferase (AST; $\widehat{\beta} = \text{-}0.023; p = 1.6e\text{-}37$) and albumin ($\widehat{\beta} = 0.007; p = 7.2e\text{-}5$) (Fig. 2h). Accordingly, finding that shorter LTL causally associated with higher AST ($\widehat{\alpha} _{LTL \rightarrow T} = \text{-}0.082; p = 3.7e\text{-}11$) and lower albumin levels ($\widehat{\alpha} _{LTL \rightarrow T} = 0.050; p = 9.1e\text{-}5$), telltales of underlying liver or inflammatory conditions. Hepatic disorders, which can lead to altered levels of AST [33], are a feature of telomere biology disorders [34]. Accordingly, we observe an association between LTL and liver fibrosis/cirrhosis ($\widehat{\beta} = \text{-}0.012; p = 1.2e\text{-}10$). However, nonalcoholic fatty liver disease, which reflects the early stages of liver disease and has GWASs with larger sample sizes, does not demonstrate any significant MR effects ($p = 0.212$). Nevertheless, these results underscore the potential role of telomere-driven cellular aging in hepatic function and/or inflammatory processes.

#### Diseases

Longer LTL correlated with decreased risk for cardiovascular and pulmonary conditions, reflecting previous findings [12, 35]. For instance, LTL had a negative causal impact on aneurysm risk ($\widehat{\alpha} _{LTL \rightarrow T} = \text{-}0.190; p = 3.0e\text{-}5$) and a positive one on forced vital capacity ($\widehat{\alpha} _{LTL \rightarrow T} = 0.072; p = 3.2e\text{-}6$). In line with that, we observed a negative correlation with risk for pulmonary diseases such as emphysema ($\widehat{\beta} = \text{-}0.013; p = 3.5e\text{-}12$) or chronic obstructive pulmonary disease (COPD; $\widehat{\beta} = \text{-}0.025; \, p = 9.0e\text{-}40$). While the MR effects of LTL on emphysema ($\widehat{\alpha} _{LTL \rightarrow T} = \text{-}0.115; \, p = 0.005$) or COPD ($\widehat{\alpha} _{LTL \rightarrow T} = \text{-}0.037; \, p = 0.012$) were concordant, they did not survive multiple testing correction. In addition to replicating a previously established correlation between short LTL and increased risk for ischemic heart disease ($\widehat{\beta} = \text{-}0.024; \, p = 6.6e\text{-}41$) [35], we also found causal evidence for the effect of LTL on ischemic heart disease ($\widehat{\alpha} _{LTL \rightarrow T} = \text{-}0.064; \, p = 3.5e\text{-}10$) (Fig. 2i–j). Hematological cancer risk negatively correlated with LTL ($\widehat{\beta} = \text{-}0.015; p = 5.8e\text{-}14$), while longer LTL correlated with kidney ($\widehat{\beta} = 0.008; \, p = 9.4e\text{-}05$) and prostate ($\widehat{\beta} = 0.029; \, p = 2.1e\text{-}23$) cancer risk. While we do not have causal estimates for the former, MR confirmed that LTL causally increased risk for kidney cancer ($\widehat{\alpha} _{LTL \rightarrow T} = 0.048; p = 8.1e\text{-}10$) and we found a near-significant trend for prostate cancer ($\widehat{\alpha} _{LTL \rightarrow T} = 0.080; p = 4.0e\text{-}4$) (Fig. 2k), aligning with previous findings [17, 20, 36, 37]. This paradox, in which both longer and shorter LTL impact disease risk, was also observed in disorders with an autoimmune component, where shorter LTL is a risk factor for rheumatoid arthritis ($\widehat{\alpha} _{LTL \rightarrow T} = \text{-}0.087; p = 6.1e\text{-}5$) and Alzheimer’s disease ($\widehat{\alpha} _{LTL \rightarrow T} = \text{-}0.038; p = 1.2e\text{-}4$), while longer LTL increased risk for systemic lupus erythematosus ($\widehat{\alpha} _{LTL \rightarrow T} = 0.167; p = 5.8e\text{-}5$) (Fig. 2l–o). Overall, these results highlight the disease-promoting role of both long and short LTL, aligning with previous findings that both shorter and longer telomeres are associated with premature death [18].

### Mediating role of LTL

**Fig. 4:**
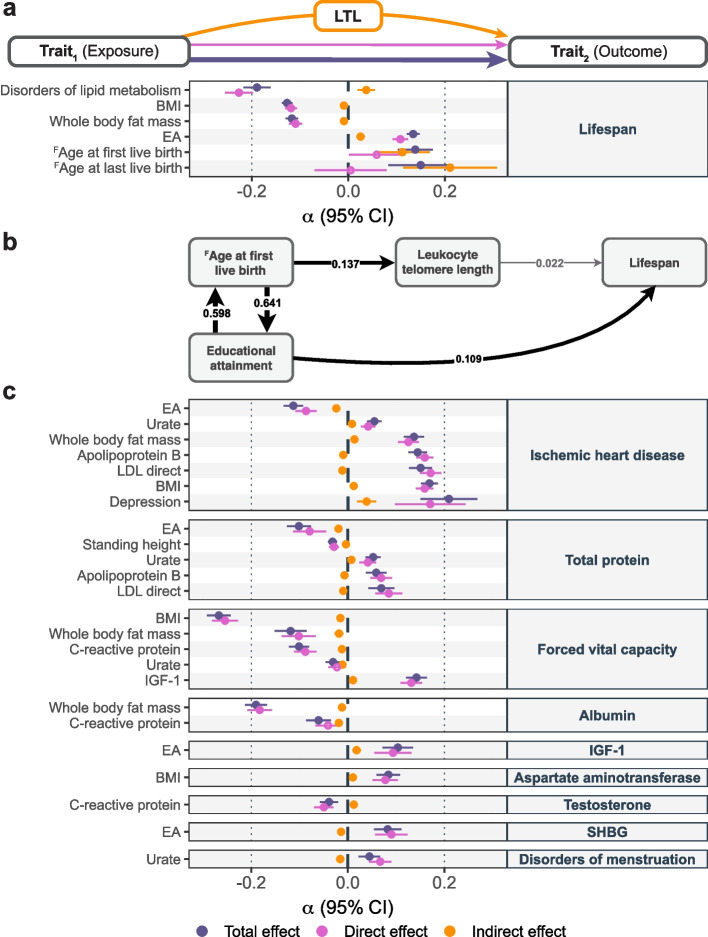
Mediating role of LTL. **a** Mediation analysis of 18 LTL-affecting exposures (*y*-axis; left) on lifespan (*y*-axis; right) through LTL with effect size estimates and 95% confidence intervals (CI; *x*-axis) of the total effect (i.e., IVW MR estimate of exposure on outcome; purple), direct effect (i.e., not mediated by LTL; MVMR estimate; pink) and indirect effect (i.e., LTL mediation by product method; orange) as displayed in the scheme on top of the figure. Displayed are relationships with significant ($p < 0.05/323 = 1.5e\text{-}4$) total and indirect effects. **b** Schematic illustration of the magnitude and direction of nominally significant MVMR effects ($p < 0.05$; gray arrow). Strictly significant ($p < 0.05/141 = 3.5e\text{-}4$) effects are shown as black arrows. Arrow thickness is proportional to the effect size. Nominally significant effect from lifespan to EA is not displayed. **c** Mediation analysis of 18 LTL-affecting exposures (*y*-axis; left) on 17 LTL-affected outcomes (*y*-axis; right) through LTL. Legend as in **a**. EA = educational attainment; LDL = low-density lipoprotein; BMI = body mass index; IGF-1 = insulin-like growth factor 1; SHBG = sex hormone binding globulin; LTL = leukocyte telomere length. Labels preceded by an uppercase F denote female-specific traits (i.e., age of first and last live birth)

Analogously to DNA methylation, LTL represents a marker of biological age that can be viewed as a clock integrating a broad range of lifestyle and health parameters [38]. This raises the question of whether LTL mediates the relation between complex traits and lifespan. We tested the mediating role of LTL for the relation between 18 non-hematological LTL-modulating traits and lifespan, the latter being affected by LTL at nominal significance ($\widehat{\alpha} _{LTL \rightarrow T} = 0.023; p = 0.008$). We identified six significant indirect effects ($p_{\text {indirect}} < 0.05/323 = 1.5e\text{-}4$), i.e., mediated through LTL (Fig. 4a and Additional file 2: Table S4). For instance, the negative impact of BMI ($P_\text{M}=7.2\%;95\%\;\text{CI = }\lbrack3.9\%;10.6\%\rbrack$) or the positive effect of EA ($P_\text{M}=18.8\%;95\%\;\text{CI = }\lbrack12.3\%;25.7\%\rbrack$) on lifespan were partially mediated by LTL. Given the considerable mediation of AFB ($P_\text{M}=80.8\%;95\%\;\text{CI = }\lbrack39.4\%;100\%\rbrack$) and ALB ($P_\text{M}=100\%;95\%\;\text{CI = }\lbrack70.1\%;100\%\rbrack$) on lifespan by LTL, we further investigated these traits through an iterative MVMR approach to build a causal network (Fig. 4b and Additional file 2: Table S3). Results emphasized the partial mediating role of LTL and EA on the effect of AFB on lifespan.

Given that lifestyle factors were found to affect LTL, which in turn influences risk for many diseases, we next used MVMR to assess the LTL mediatory effect for all pairs of 18 LTL modulators and 17 LTL-affected traits. We identified 24 significant ($p_{\text {indirect}} < 0.05/323 = 1.5e\text{-}4$) LTL-mediated relationships (Fig. 4c and Additional file 2: Table S4). Effects on ischemic heart disease, total protein levels, and forced vital capacity were the most frequently mediated by LTL, whereas urate levels, CRP, BMI, BFM, and EA were the most common exposures. Overall, while we do detect a substantial number of significant mediations through LTL, the average mediation proportion is 5.71%, only accounting for a fraction of these relations.

## Discussion

In this study, we comprehensively examined the bidirectional causal relationships between LTL and complex traits, diseases, and lifestyle factors and used MVMR to examine causal effect mediation. Our study reiterates age and sex as major determinants of LTL variability [18, 22] and confirms the causal effects of lifestyle factors on LTL. Furthermore, we provide robust evidence for a causal role of abnormal LTL in a broad spectrum of clinically relevant traits, including cancer, autoimmune disorders, lung diseases, and cardiovascular conditions. Lastly, our results show that LTL partially mediates the effect of BMI, EA, and reproductive traits on lifespan.

Others [20, 21, 39] have used MR to estimate the impact of LTL on the human phenome. In contrast to Codd et al. [20], we used summary statistics originating from large consortia, which offer the double advantage of reducing sample overlap between the exposure and outcome sample and have much higher case numbers (and thus power) than in the relatively healthy UKBB. We hypothesize that this explains some of the nine novel findings that are unique to our study, five of which were tested but did not yield significant results in Codd et al. (Additional file 2: Table S5). For instance, our results support the Alzheimer’s disease risk-increasing effect of shorter LTL, which was proposed to be driven by promotion of cellular senescence [40–45]. In line with previous literature [46, 47], our results also suggest that longer LTL increases the risk of systemic lupus erythematosus. Combined with our confirmation that shorter LTL raises rheumatoid arthritis risk, these findings highlight the implication of LTL in autoimmune conditions. Finally, we report a coherent impact of longer LTL on risk for menstrual disorders [21], once again linking LTL to female reproductive health (Fig. 3). Overall, this supports the deleterious role of both long and short telomeres in human health.

Unlike previous large-scale studies investigating the link between LTL and the human phenome [18, 20, 37, 39], we also estimated the causal effects of phenotypes on LTL. In line with prior research, alcohol consumption [48], smoking [8, 49], obesity [50], and socio-economic disadvantages [51, 52] emerged as significant contributors to telomere shortening, underscoring the potential benefits of lifestyle modifications. Some of these factors, such as BMI and EA, were found to exert a small, albeit significant proportion of their impact on longevity through LTL. Our findings further indicate that the impacts of both depression and urate levels on LTL are partly exerted through CRP, highlighting inflammation’s role as a mediating factor of LTL shortening [29]. Surprisingly, the positive influence of serum lipid levels on LTL often attenuated the total effect of lipid-trait relationships. These results are unexpected as high cholesterol levels promote oxidative stress [53], which in turn accelerates LTL shortening [54]. Yet they are coherent with recent literature [55] suggesting a nuanced protective role of specific lipids and lipoproteins in maintaining telomere length. However, our inability to replicate these effects when using independent LTL summary statistics, possibly due to lack of statistical power, warrants further investigation into the mechanisms through which elevated lipid levels may support telomere preservation.

One of the most intriguing findings of our study is the causal relationship between delayed AFB and ALB and extended LTL in females, which was only partially confounded by SES. These results align with the accelerated LTL shortening rate we observed after childbirth, which is further exacerbated post-menopause. Although we did not observe an association between oestradiol levels — measured only in $\sim$49,000 UKBB female participants — and LTL, we hypothesize that hormonal shifts following pregnancy and menopause could accelerate LTL shortening [56]. An alternative explanation is that LTL shortening is driven by the stress imposed by such events on the body, aligning with literature that posits pregnancy as a significant accelerator of biological aging, measured per methylation [57]. Notably, no significant association between age at menarche and LTL was found. While this could reflect a genuine absence of association, inaccurate reporting of age at menarche and inability to assess shifts in telomere shortening rate before adulthood [30] could also contribute to this negative result. Extending prior literature [58], our findings suggest that delayed female age at childbirth increases longevity partly through telomere length, even though we cannot exclude partial confounding by SES, which also significantly influences this relationship. While further research is required to test these hypotheses, our results highlight the prominent role of life history events in LTL shortening rates.

Our study is subject to several limitations. First, the use of cross-sectional bulk LTL limits our capacity to analyze individual telomere shortening rates, which might be a critical factor in disease prediction [38]. Second, although LTL and telomere length in other tissues are correlated [59], this proxy might miss more subtle and tissue-specific relations between telomere length and the phenome. Importantly, the causal relevance of LTL in non-blood-related diseases may be limited, as observed associations could reflect shared genetic effects on telomere length across different tissue types, rather than direct effects of telomere length in leukocytes. Furthermore, we cannot exclude that since telomere length was measured in leukocytes, it made finding associations with hematological traits more likely, despite our sensitivity analyses showing robustness against confounding by hematological factors. Additionally, our study focused on White-British ancestry, meaning the results may not translate to other ancestral groups. In the future, single-cell telomere length measured at chromosomal resolution through long-read sequencing approaches across various tissues, time points, and ancestral groups should provide a more refined view of telomere dynamics. Third, Lasso regression in our study was limited to data without missing values. Comparisons between cases with and without missing data showed shorter mean LTL in the former, indicating that data missingness, previously correlated with several health traits [60], constitutes an additional limitation to our findings. Fourth, MR presents with inherent limitations, notably susceptibility to horizontal pleiotropy violations, especially given the considerable heterogeneity across our IVs. While MVMR analyses can mitigate biases introduced by pleiotropy and elucidate direct causal effects, these analyses are also more likely to be subject to weak instrument bias, which is indicated by several conditional F-statistics falling below 10 (Additional file 2: Table S3) [61]. We used a broad range of sensitivity analyses and focused on results robust across these various methods. Another limitation of MR is that detection power is bound by the number of available IVs, so that our power to detect causal relations between traits and LTL is variable across phenotypes and might be lower or larger than for the reverse LTL on trait relation, depending on whether the trait has less or more IVs than LTL, respectively. Moreover, we derive our causal estimates from two unidirectional models, each fitting a different causal direction. Not using an explicit bidirectional model may marginally overestimate the effect sizes for the five hematological traits with bidirectional MR effects, but the expected bias is minimal [62]. Fifth, treating diseases as binary exposures may violate the exclusion restriction assumption [63], prompting careful interpretation of the effects of hypertension, lipid disorders, and depression on LTL. Finally, MR does not account for dynamic spatiotemporal changes in LTL that occur over lifetime and/or in the context of some diseases such as cancer.

## Conclusions

In conclusion, through usage of rigorous univariable and multivariable bidirectional Mendelian randomization, we identify a complex network of causal relations wherein both exogenous (i.e., lifestyle or environmental) and endogenous (i.e., physiological) factors modulate LTL, which in turn influences the risk for numerous diseases and mediates the impact of some of these traits on lifespan. Still, based on currently available data, the mediating role of LTL between lifestyle and disease risk is estimated to be modest, and further research is needed to explore the relation between LTL and other aging biomarkers, such as DNA methylation, to understand its clinical value as a proxy of biological age.

## Methods

### Software

All analyses were conducted using R v4.2.1 and Python v3.11.3. PLINK v1.90b7 [64] was used. Workflow management was facilitated by Snakemake v7.25.3 [65].

### Data

#### Individual-level UK Biobank data

Observational analyses were carried out in the UK Biobank (UKBB), a cohort of $\sim$500,000 volunteers from the general UK population aged between 40 and 69 years at recruitment [66]. Analyses were conducted on 326,363 participants with known sex, age, and LTL after the exclusion of individuals of non-white and non-British ancestry (self-reported + genetically defined), relatives ($\le 3^{rd}$ degree), and gender mismatches (see UKBB Resource 531), as well as those who retracted their participation. Given that LTL measurements are derived from blood, we further excluded 4,376 individuals with blood malignancies, based on self-reports (UKBB field #20001 codes 1047, 1048, 1050, 1051, 1052, 1053, 1055, 1056, 1058) or hospital diagnoses (#41270; International Classification of Diseases 10^th^ Revision [ICD10] codes mapping to the Phecode “cancer of lymphatic and hematopoietic tissue” [67]).

We used technically adjusted and standardized LTL (#22192) [19] and assessed its relation to 166 complex traits (Additional file 2: Table S1). These include 60 common diseases defined based on hospital diagnoses (#41270; last diagnosis September 2021), while excluding from controls individuals with self-reported (#20001, #20002) or hospital-diagnosed (#41270) conditions related to the investigated disease [68]. Disease phenotypes were used to calculate a disease burden phenotype, i.e., the total number of diseases diagnosed in an individual among the 60 considered ones. The remaining 105 traits include 11 anthropometric traits (e.g., weight), 41 biomarkers (e.g., serum lipids), 18 life events (e.g., age at menarche and menopause), 26 lifestyles (e.g., beef intake) and socio-economic factors (e.g., Townsend deprivation index), and 9 miscellaneous traits. Definitions of composite phenotypes are described in the Additional file 1. Briefly, continuous traits with multiple instances were averaged, while the first instance was used for integers or factors. To minimize noise, outliers (mean ± 5 standard deviations [SD]) in continuous traits were removed. Factorial variables were numerically converted for efficient integration into the regression model. All traits, including binary predictors, were then scaled to have zero mean and unit variance to obtain more comparable effect sizes. As the 167 assessed traits (i.e., 166 above-mentioned + blood cancer) were partially correlated we estimated the number of effective tests [69], i.e., the number of tests needed to explain 99.5% of the variance in our phenotypic dataset, to 141, resulting in a significance threshold of $p < 0.05/141 = 3.5e - 4$ for observational correlation and MR analyses.

#### GWAS summary statistics

When available (i.e., for non-composite traits), genome-wide association study (GWAS) summary statistics originate from the Neale group (file release July 2018 [70]; Additional file 2: Table S6). Summary statistics for reproductive lifespan were derived from GWAS on age at menopause and menarche by first back-transforming the effects on year-scale and then computing their difference:

$$\begin{aligned} \beta= & {} SD_{menopause} \times \beta _{menopause} - SD_{menarche} \times \beta _{menarche} \\ SE= & {} \sqrt{SD_{menopause}^2 \times SE_{menopause}^2 + SD_{menarche}^2 \times SE_{menarche}^2} \end{aligned}$$

The sample size for the resulting summary statistic was set to the lowest of the two (i.e., age at menopause; $N = 111,593$) and *p*-values were computed with a two-sided test based on a t-statistic obtained by dividing the effect size by its standard error. For diseases, a set of previously compiled GWAS summary statistics [71] of predominantly European-descent consortia meta-analyses was used (Additional file 2: Table S6). CHIP summary statistics, originally in build 38, were mapped to human genome build 37 using UCSC LiftOver [72]. Summary statistics were harmonized with the UK10K reference panel [73] and restricted to autosomal chromosomes. After excluding palindromic single-nucleotide polymorphisms (SNP) and adjusting strand-flipped SNPs, effect sizes were standardized to represent the square root of the explained variance [74].

### Observational correlation

#### Predictors of LTL variability

To estimate the fraction of LTL variability explained by the human phenome, we used Lasso regression (glmnet package in R [75]) with unadjusted normalized LTL as the outcome variable and traits with less than 5%, 7%, and 10% missing data as possible predictors in a joint model. Given the non-deterministic choice of the optimized regularization parameter (one SE rule lambda), 50 regressions were fitted and traits that were selected in at least 95% of the cases were considered as predictors.

#### Single trait linear regression

We adjusted LTL by regressing out age, age^2^, genotyping array, sex, and the interaction of the latter with the priors as fixed effects and used this variable as the outcome in 166 linear regression models with the traits described in Additional file 2: Table S1 as explanatory variables. Effect sizes reported in text are in $\text {SD}_{\text {LTL}}\text {/SD}_{\text {Trait}}$, except for the effect of age, in which case effects are reported in $\text {SD}_{\text {LTL}}$/year. We followed up on specific associations with sensitivity analyses to identify possible confounders:

- In individuals using cholesterol-lowering drugs (#6177 and #6153), serum lipid levels were corrected for average simvastatin effect, i.e., + 1.6 mmol/L, 1.4 mmol/L, 0.4 mmol/L, − 0.1 mmol/L of total cholesterol, low-density lipoprotein (LDL), triglycerides and high-density lipoprotein (HDL), respectively [76].
- Reproductive traits showing significant ($p < 0.05/141 = 3.5e\text{-}4$) association with LTL were corrected for socio-economic status (i.e., Townsend deprivation index (#189), average total household income before tax (#738), and educational attainment (see Additional file 1)).
- In addition to age, sex, and array, LTL was corrected for eosinophil (#30150), lymphocyte (#30120), monocyte (#30130), neutrophil (#30140), platelet (#30080), red blood cell (#30010), reticulocyte (#30250), and white blood cell (#30000) counts and linear regressions with non-blood trait count traits were performed anew to ensure the LTL associations were unbiased. As a result, the available sample size was reduced to $N = 308,346$.

#### Female reproductive phases

To assess the impact of childbearing and menopause on LTL, we identified three distinct female reproductive phases: (1) years before first live birth, (2) premenopausal years after first live birth, and (3) postmenopausal years. Number of years spent in each phase was derived from current age (#21003), age at first live birth (#2754), and age at menopause onset (#3581). Phases (2) and (3) were set to 0 for females with no children (#2734: number of live births $= 0$) and premenopausal women, respectively. The joint linear regression model included time spent in each phase and two indicator variables for whether the women carried a pregnancy to term and experienced menopause. Female participants who had their first child post-menopause, lacked a menopausal status (#2724) or age at menopause (#3581), or did not specify childbirth events (#2734) or age at first childbirth (#2754) were excluded from this analysis.

### Mendelian randomization

#### Bidirectional univariable Mendelian randomization

GWAS summary statistics were used to conduct bidirectional two-sample MR, with $\widehat{\alpha} _{LTL \rightarrow T}$ representing the causal impact of LTL (exposure) on complex traits (outcome) and $\widehat{\alpha} _{T \rightarrow LTL}$ the causal impact of complex traits (exposure) on LTL (outcome) (Fig. 1). Harmonized SNPs significantly associated ($p < 5e\text{-}8$) with the exposure were clumped ($p1 = 0.0001$, $p2 = 0.01$, $kb = 250$, and $r2 = 0.01$) with PLINK v1.9 [64] and retained as instrumental variables (IVs). As the *HBB* gene was used as a control for the LTL measurements, SNPs in this gene (chr11:5,246,696–5,248,301; GRCh37/hg19) that associated with LTL were removed to prevent spurious associations [20]. For LTL IVs, this led to the exclusion of a single variant, rs1609812. Due to the complex long-range linkage disequilibrium (LD) structure of the HLA locus, SNPs mapping to that region (chr6:25,000,000–37,000,000; GRCh37/hg19) were also excluded from our IVs [77]. For each exposure-outcome pair, further IVs were removed based on difference in allele frequency ($\ge 0.05$) and Steiger filtering ($Z \le \text{-}1.96$). Bidirectional MR analyses were carried out with the TwoSampleMR R package (v0.5.7) [78], primarily through the IVW method. LTL on trait and trait on LTL MR effects were computed for 152 and 142 traits, respectively, with at least two IVs.

Sensitivity analyses for relationships with significant IVW MR effects were conducted using additional MR methods, i.e., MR Egger, simple mode, weighted median, and weighted mode, to ensure robustness of the results. Heterogeneity was assessed using Cochran’s Q-statistics. Given a high proportion of elevated Q-statistics, we additionally run MR-PRESSO [79] for relationships with significant IVW MR effects. To further ensure that our results are not biased by pleiotropy – which violates the MR assumption that IVs only affect the outcome through the exposure [80] – we first filtered genome-wide significant exposure SNPs and harmonized these SNPs across all 153 traits with available GWAS summary statistics, i.e., verified that SNPs are present across the 152 traits + LTL summary statistics. This step is carried out before clumping to guarantee that the identified IVs are consistently present across all outcomes, enabling subsequent comparisons. After clumping, Steiger filtering was applied between the exposure and all other traits to ensure that the selected SNPs are more strongly associated with the exposure than with any of the other included traits. SNPs that passed filtering for all traits were retained as IVs and MR analyses were conducted on these. This approach serves as a reasonable pleiotropy filter due to the diverse nature of our phenotypes. While sample overlap in two-sample MR may bias results toward observational effects, no overlap may also lead to ’winner’s curse’ bias [81, 82]. Weak instruments can further exacerbate these biases. Although our extensive simulations have demonstrated that these issues lead to mild biases [82], we used MR-APSS [83] (default parameters and LD scores from 1000 Genomes Data [84]), which addresses both sample overlap and pleiotropy. Finally, we replicated the MR analyses using LTL summary statistics generated based on an independent sample ($N = 62,271$) from the biobank of Vanderbilt University Medical Center (BioVU) and Marshfield Clinic’s Personalized Medicine Research Project (PMRP) [18, 85, 86].

#### LTL mediation analysis

Excluding hematological traits due to potential confounding, we used a two-exposure multivariable MR (MVMR) framework to individually assess the mediating role of LTL across 18 LTL-affecting traits ($p < 0.05/141 = 3.5e\text{-}4$) on lifespan, proxied through parental lifespan [87]. We further examined the global mediatory role of LTL between each of these 18 LTL-affecting traits and 17 traits causally impacted by LTL ($p < 0.05/141 = 3.5e\text{-}4$). This corresponded to 323 pairs (18 * 18 (i.e., 17 traits + lifespan), excluding one pair as IGF-1 associated with LTL as both exposure and outcome). This sets our significance threshold for the total and indirect effects at $p < 0.05/323 = 1.5e\text{-}4$. IVs for mediation analyses were selected from summary statistics through a two-phase clumping process (see the “Bidirectional univariable Mendelian randomization” section) in which harmonized exposure IVs (i.e., trait IVs) were first independently clumped. In the second phase, exposure and mediator IVs (i.e., LTL IVs) were clumped together, prioritizing the former over the latter (i.e., retaining exposure IVs over mediator IVs). Providing MR assumptions hold, by instrumenting both the exposure and mediator we also reduce confounding bias between mediator and outcome. Steiger filtering was applied to both exposure IVs with respect to outcome and mediator and to mediator IVs with respect to the outcome. Indirect effects were determined through two strategies: difference in coefficients and product of coefficients [88]. The former subtracts the direct effect (MVMR) from the total effect (IVW), while the latter multiplies the univariable MR estimates from the exposure on the mediator by the MVMR effect of the mediator on the outcome. Both approaches generated consistent results (Additional file 1: Fig. S9) and we present the product of coefficients method due to easier interpretability in the main text. We further corrected these estimates for regression dilution bias [74]. Mediation proportions ($P_{\text {M}}$) represent the ratio of the indirect ($\alpha _{\text {indirect}}$) to total ($\alpha _{total}$) effect with 95% confidence intervals (upper limit capped at 100%) estimated from the 2.5th and 97.5th quantiles of the distribution of 10,000 simulated ratios drawn from $\tilde{\alpha }_{\text {indirect}} \sim N\left( \hat{\alpha }_{\text {indirect}}, \hat{\text {SE}}(\hat{\alpha }_{\text {indirect}})\right)$ and $\tilde{\alpha }_{\text {total}} \sim N\left( \hat{\alpha }_{\text {total}}, \hat{\text {SE}}(\hat{\alpha }_{\text {total}})\right)$.

#### Multi-trait analysis for direct effect estimation

For MVMR with multiple exposures and no predefined mediator, IVs were selected through a two-step process [89]. First, SNPs for each exposure were ranked according to their *p*-values (more significant *p*-values receiving lower ranks) and minimum rank across all exposures was determined for each SNP. This minimum rank was used to prioritize SNPs in a subsequent clumping process. IVs were filtered as previously described. Finally, MVMR regression estimates were compared to univariable MR estimates (see the “Effect size comparison” section). For the univariable MR, we either used the same IVs as in the MVMR or employed a subset of IVs, which were retained after Steiger filtering between both the outcome and the exposure of interest, as well as between the exposure of interest and the other exposures. We report weak instrument bias via conditional F-statistics [61] and heterogeneity through Cochran’s Q-statistic [90] (Additional file 2: Table S3).

### Effect size comparison

Significant differences between two estimated effect sizes $\widehat{\beta }_{\text {X}}$ and $\widehat{\beta }_{\text {Y}}$ were assessed with a two-sided *p*-value ($p_{\text {diff}}$) derived from:

$$\begin{aligned} t = \frac{{\widehat{\beta }_{\text {X}} - \widehat{\beta }_{\text {Y}}}}{{\sqrt{SE_{\text {X}}^2 + SE_{\text {Y}}^2}}} \end{aligned}$$

which assumes that the two estimates are uncorrelated. Often these estimates have a positive correlation (as estimated from the same data) and hence the *t*-statistic has a variance smaller than one, thus the test is conservative. This approach was used throughout the study to assess the effect of sensitivity analyses and compare univariable MR and MVMR results.

### Supplementary Information

Additional file 1: Supplementary note. Definition of composite traits. Fig. S1. Linear regression of LTL against age, stratified by sex. Fig. S2. Decomposition of the effects of mother’s and father’s age at birth on LTL. Fig. S3. MR estimates of LTL on traits across different methods. Fig. S4. MR estimates of trait on LTL effects across different methods. Fig. S5. Stringent Steiger pleiotropy-sensitivity analysis. Fig. S6. Linear regression estimates adjusted for blood counts. Fig. S7. LTL associations adjusted for potential confounding variables. Fig. S8. MVMR effect estimation of traits on LTL. Fig. S9. Mediating role of LTL on complex trait pair relations.

Additional file 2: Table S1. Description of analyzed complex traits. Table S2. Associations of traits with LTL: observational and MR estimates. Table S3. Complementary LTL association analyses. Table S4. Mediation analysis results. Table S5. Comparative analysis of LTL on traits MR findings. Table S6. GWAS summary statistics sources.

Additional file 3: Review history.

## Data Availability

UKBB data are available for registered users. UK10K reference panel is available upon request at https://www.uk10k.org/data_access.html [73]. European LD scores from 1000 Genomes Data are freely accessible [84, 91]. GWAS summary statistics originate from various sources: Alzheimer’s disease [92], lifestyles [93], sleep apnea, celiac disease, endometriosis, pneumonia, psoriasis, and valvular heart disease [94], asthma [95], balding [96], breast cancer [97], bipolar disorder [98], cataract [99], CHIP [26], liver fibrosis [100], CKD [101], atrial fibrillation [102], colorectal cancer, kidney cancer [103], depression [104], epilepsy [105], glaucoma [106], inflammatory bowel disease [107], coronary artery disease [108], kidney stones [109], multiple sclerosis [110], osteoarthritis [111], ovarian cancer [112], prostate cancer [113], Parkinson’s disease [114], lifespan [87], rheumatoid arthritis [115], schizophrenia [116], sex [117], smoking cessation [118], stroke [119], type 1 diabetes [120], WHR [121], LTL [18, 20], and else from the Neale Lab [70] or Pan-UK Biobank [122]. Links to download the summary statistics are provided in Additional file 2: Table S6. Code used in this study is available under the Creative Commons Attribution 4.0 International License (CC BY 4.0) on GitHub [123] or on Zenodo [124].
