# Supplementary material for: Breaking down causes, consequences, and mediating effects of telomere length variation on human health
Source: Genome Biol. 2024 May 17;25:125. doi: 10.1186/s13059-024-03269-9 (PMC11101352; doi:10.1186/s13059-024-03269-9)
Supplement: Supplementary file 3 — Additional file 3: Review history. [file 13059_2024_3269_MOESM3_ESM.docx]

**1^st^ round**

**Reviewer 1**

This manuscript reports an investigation of the causes and consequences inter-individual variability in leukocyte telomere length. Using data from the UK Biobank, the authors conduct bidirectional Mendelian randomization analyses, identifying numerous diseases, traits, and biomarkers that show evidence of affecting and/or be affected by LTL. Overall, the work is rigorous and comprehensive, and the paper is well-written. The primary limitation of this paper is the limited novelty. While there are a few novel associations reported here, the majority of the results are confirmatory, having previously been reported, either based on UK biobank data or independent data sets. A few additional concerns are described below.

ABSTRACT
--"we show that LTL partially mediates the impact…" This is worded too strongly, MR assumptions cannot be proved and lifespan is not measured directly.

RESULTS
--Why is mother and father's age (at participant birth) analyzed in the "complex trait" section? Should those not be considered predictors of TL and put in the prior section?

--Fig 3: in my view the figure is a bit misleading. It is well know that LTL shortening is most rapid during development and early life (when stem cells are dividing rapidly to support growth and development). Also there is no actual data prior to age 40, so the trajectory shown from age 0 to 70 seems to send the wrong message. At least on first look. Also, technically "shortening" should not be used in the title, as you're looking at the association between LTL and age, not shortening among participants. Also the meaning of "predicted LTL" vertical axis label is unclear.

--Fig 4. Need to make clear which analyses correspond to women only. I assume anything involving AFB or ALB, what about the rest of the figure?

METHODS
-- "As the HBB gene was used as a control for the LTL measurements [20], we removed the single SNP (rs1609812, p = 3.9e−65) associated with this gene (chr11:5'246'696-5'248'301; GRCh37/hg19) to prevent spurious associations." This text needs some additional explanation. And the P-value corresponds to what?

DISCUSSION
--Seems unlikely to me that TL in leukocytes plays a causal role in non-blood cancers. More likely to be TL in the cancer-relevant tissue, with some SNP effects on TL being shared across tissue types. This could also be true for other diseases, such as lung diseases/traits. This possibility warrants some discussion, as blood cells will not always be the causal tissue/cell type

--If your MVMR approach for mediation analysis susceptibility to bias from mediator outcome confounding? Clarify for the reader, as confounders (U) and IV/SNPs for MVMR/mediation analysis are not shown in Figure 1.

--Authors should acknowledge the prior literature on educational attainment at TL in UKB and integrate into the discussion/interpretation of their analyses involving EA (Amin et al 2022). There are also other prior UKB papers that investigate TL in relation to cardiovascular disease, alcohol, modifiable traits/behaviors, and other phenotypes using MR. These should also be integrated into the discussion.

**Reviewer 2**

The manuscript by Moix and colleagues explores the causal relationship between leukocyte telomere length (LTL) and various phenotypes using Mendelian randomization (MR). The study employs instrumental variables (IV) derived from summary statistics of a published GWAS study to proxy LTL levels. The investigation into the associations between LTL, a marker of aging, and diverse phenotypes adds an interesting dimension to the field. However, the paper requires substantial revision before it can be considered for acceptance.

Major Comments:

1. Sample Overlap in Two-Sample MR:
The authors claim to have used two-sample MR methods, but it is noted that all summary statistics are derived from the UK Biobank data. Sample overlap in two-sample MR may introduce bias (Burgess et al. 2016, PMID: 27625185). While Minelli et al. (2021, PMID: 33899104) tested the validity of some common 2-sample methods in a biobank-scale study, but they also indicates that method like MR Egger is biased. it is also unclear if this extends to the specific methods used in the present study (e.g., MR-PRESSO and MVMR). The authors should either validate their methods for sample overlap or ensure independence between exposure and outcome GWAS samples.

2. Clarification of Analysis Methods:
It is unclear in methods section whether mediation analysis and MVMR were performed using individual-level data with one-sample methods or summary statistics with two-sample MR methods. The software or packages used for these analyses need to be specified.

If summary statistics and two-sample MR methods were used (as illustrated by Figure 1), potential biases due to sample overlap need to be addressed. Alternatively, one-sample MR using individual-level data should be considered, with a clear specification of how covariance between the effects of genetic variants on each exposure is treated (Sanderson et al., 2019, PMID: 30535378).

Given these uncertainties in the methodology and study design, it is advisable not to draw definitive conclusions from the results until the aforementioned aspects are clarified. The potential for bias in the results needs to be addressed before meaningful interpretations can be made.

3. Reference 20 and Novelty of Findings:
Reference 20 (Codd et al.) has previously performed one-sample MR between LTL and various traits and diseases (93 quantative traits and 123 diseases). The authors should clarify if they found any novel associations when considering LTL as an exposure. Emphasizing the novelty of findings, if present, is crucial to distinguish this research from previous studies.

4. Power and Weak Instrument Bias:
The sample sizes of cases for many diseases are low, potentially resulting in a low percentage of variance explained by instrumental variables. F-statistics for all IVs for each phenotype in univariable MR should be provided to demonstrate that the MR is not suffering from weak instrument bias (not just conditional F-stat in MVMR). The authors should discuss the power of MR analyses given the current sample size.

5. Bidirectional MR and Violation of Assumptions:
In bidirectional MR, treating diseases as binary exposures may violate the exclusion restriction assumption. The authors should evaluate and discuss the validity of the method and the interpretation of results in light of this potential limitation (Burgess et al., 2018, PMID: 30039250).

Minor comments:

1. MR Egger Intercept:
The manuscript should include the intercept of MR Egger regression, not just MR estimates. This provides an indication of pleiotropy when non-null.

2. Exclusion of SNPs in HLA Locus:
The removal of SNPs in the HLA locus from instrumental variables is mentioned in the method section. However, the authors should consider that these SNPs could be significantly associated with certain traits or diseases (e.g., rheumatoid arthritis) and their exclusion may compromise the power of MR.

In summary, addressing these major comments is crucial for ensuring the robustness and validity of the study's findings. Substantial revisions are recommended before the manuscript can be considered for acceptance.

**Reviewer 3**

Moix et al use Mendelian Randomisation to investigate the associations, causes, consequences of variation in measured leukocyte telomere length using UK Biobank data. Their findings will be of interest to those studying telomere biology, ageing and age-associated diseases. The statistical approaches and choice of covariates appear appropriate. Interpretation of results is mostly appropriate, with some exceptions. Below I have some suggestions aiming to further improve the manuscript.

Major
1. The authors need to consider more carefully that leukocyte telomere length (LTL) is a measurement of telomere length of a diverse mixture of leukocytes and can be affected by somatic mutations: In particular, the phenomenon of clonal hematopoiesis has been variously found to be associated with non-hematological diseases, including cardiovascular disease. Clonal hematopoiesis is also associated with greater attrition of telomere length and this may be the basis for the association between lower LTL and hematological cancer (as clonal hematopoiesis is the precursor to many blood cancers).
The authors need to consider the above and ensure their analysis takes it into account. They should at least repeat their analyses after excluding participants with clonal hematopoiesis. It may be also informative to examine the interaction of clonal hematopoiesis and LTL in driving diseases, but admittedly this will not be straightforward.

Minor
1. Table 1: as this is a MR study, it would be better to avoid using the term "determinants" of LTL for these observational analyses and use terms like "associations" (saving terms that imply causality for MR analyses)
2. The reasons for the association of paternal age with LTL may be very different to those for maternal age. As paternal and maternal age corelate between them, it would be interesting to understand the degree of independence of the association of LTL with paternal vs maternal age (e.g. by conditioning one or other).
3. iIt is recognized that lymphocyte telomere length differs from neutrophil LTL. In this light, the observed associations of LTL with lymphocyte, white blood cell, monocyte, eosinophil and neutrophil counts may simply reflect these differences in telomere length between myeloid and lymphoid cells, rather than a "causal" consequence of telomere length. This should be discussed. Also including these blood counts as covariates may change some of the results pertaining to other diseases, and should be attempted.
4. Line 218: the discussion about depression causing oxidative stress may be a bit simplistic, particularly as it ignores known links between depression and other factors that affect LTL including many identified within this manuscript (e.g. physical activity, diet, smoking, alcohol intake, WHR, social habits etc)
5. Line 233 onwards: consider changing "birth" to "childbirth", to avoid readers thinking that the former refers to the participants birth or factors related to it (e.g. prematurity, birth weight etc). Also, "Figure 3" should say "Age at first (child)birth" not "Age of first birth".
6. In relation to telomere attrition in women and childbirth/menopause, It is not clear of the authors had information about the age at menarche. If they do, it may be helpful to examine its relevance, as women with early menarche were reported to have children slightly earlier than those with later menarche in some studies.
7. Under "renal health" the authors implies that the association of Urate and LTL implies a link between LTL and renal function, but urate is not a great measurement of the latter (in line with this, much of the effect of urate on LTL is linked to high CRP). Cystatin C is a good measure if renal function, but can also be affected by inflammation. It would be good to comment on the impact of CRP on the cystatin-LTL association and also on any link between creatinine and LTL (creatinine is a less good measure of renal function than cystatin when there is no inflammation, but probably a better one when there is). If creatinine is not associated with LTL, then it may be unsafe to link LTL with renal function.
8. Under "hepatic biomarkers" the authors should consider that hepatic disease is a feature of telomere biology disorders, as is pulmonary fibrosis, and add some relevant discussion.
9. The authors should also discuss the fact the LTL is a measure of blood cell telomeres and whilst this corelates with telomere length in other tissues, this correlation is not perfect. This is relevant to the fact that the tissue-specificity of telomere biology disorders (where the telomere length of individual tissues may be a stronger determinant t of phenotypes) is not the same as the diseases linked to LTL.

**Reviewer 4**

Review of GBIO-D-23-01573 Moix et al., "Breaking down causes, consequences, and mediating effects of age-related telomere shortening on human health"

Overall, this is a strong paper using Mendelian randomization to examine the role of leucocyte telomere length (LTL) on a variety of serious health problems that potentially affect lifespan. It uses the UK biobank data on telomere length of almost a third of a million research participants. This large dataset offers substantial statistical power to detect relatively small effects between genomic instrumental variables and health outcomes that are potentially mediated via LTL. Most of the comments I have are minor corrections or requests for more details or clarification.

An important aspect of this paper is that it explicitly considers bidirectional causation between LTL and health outcomes. Thus, the authors identify 23 significant causal effects of traits on LTL and 26 significant effects of LTL on traits. These estimates have been obtained by fitting two unidirectional models, one in each causal direction. While these are unlikely to be distant from those that would be obtained from an explicit bi-directional causal model. Several such models have been described in the literature, so it seems reasonable that they should be employed in the present case.

In common with many such investigations, the Type I error rate was not corrected for once the sample was subsetted into males and females. To mitigate this, the Bonferroni approach is pretty conservative, so it's probably not a big deal, but should be addressed by the authors.

Details:

p.6 line ~53:
Setting non-parous and postmenopausal women's scores to zero for number of years is an unusual choice, since these data are effectively missing, and should not be scored as zero. It would help to revise this coding and report the effect of so doing on the parameter estimates. Hopefully, not much will change, but estimates and their standard errors seem likely to be less biased by fitting a truly bidirectional structural equation model (SEM).

p.6 line 98:
Some fairly large proportions of the sample were lost due to regular regression's weakness that no missing observations should be present for the independent variables. This issue would be better handled via SEM, for which likelihoods can be calculated for participants with partially missing data. If this approach is not followed, then at least the means and variances of participants who were excluded should be presented alongside those who were not. Doing so will enable checking to see if the missing data points are missing completely at random, or if some other missingness mechanism is at play whose effects should be controlled.

p.6 line 100:
How were the effects of array treated? Two methods spring to mind: dummy coding yes or no for each array that was used, to end up with as many covariates as there were arrays, minus one. Another would be to estimate their influence as a random effect. It is important to clarify these details to give others even a slight chance of reproducing the results.

p.7 line 126:
Why were the additional IVs removed? Some greater clarity is needed with the steps that eliminated data; doing so can greatly affect results and reproducibility.

p.7 line 137:
Similar to prior comment, "we first 137 filtered genome-wide significant exposure SNPs and harmonized these SNPs across all 152 traits" is not clear how the harmonization was done.

p.16 top:
"Short LTL correlated with increased risk for cardiovascular and pulmonary conditions, reflecting previous findings
289 [12, 48]." This is an accurate statement, but readability is adversely affected because the descriptor "short" reverses the sign of the associations reported in the following sentences. I think it would be better to state it the other way around, that long telomeres are protective, having less adverse outcomes, then the signs of the coefficients agree with the statement.

p.21 line 361:
The word "variation" is the wrong one for this context. It isn't that variation in LTL increases risk, it's the value of an individual's LTL 'score' that does so. Variation might mean within person or across persons, and in neither case is it consistent with what is reported in the paper, i.e., one score per person.

p.22 line 378:
is should be are; data are plural.

p.22 line 388:
It would strengthen the paper to note whether parity per se is associated with longevity. In general, the discussion seems to be few connections to the prior literature, or even that which was discussed in the introduction.

p.22 line 394: "Furthermore, we cannot exclude that the tissue in which telomere length was measured (i.e., leukocytes) biases the observed associations with hematological traits, even if accounting for blood cell type traits did not seem to affect association estimates between LTL and other traits." I think the sentence would work better rewritten as follows: "Furthermore, we cannot exclude that since telomere length was measured in leukocytes, it made finding associations with hematological traits more likely. This inequality between traits may exist even if accounting for blood cell type traits did not seem to affect association estimates between LTL and other traits."

p.23: line 409:
"exogenous and endogenous environmental factors" the distinction between these types of environmental factor first appears here in the conclusion. Either define them here, or define them earlier, but interested readers are likely to want to know which are which.

Supplemental Figure 7:
"a) socio-economic status or b) cholesterol medication" in the caption does not agree with the figure itself, in which a) and b) are reversed.

**Reviewer 5**

This study presents a comprehensive analysis on the relationships between leukocyte telomere length (LTL) and 141 complex trait that are closely related to human health.

Mendelian Randomization (MR) and MVMR are used as the main method for the analyses and have revealed several interesting results. My specific comments on the methods and results are as follows:

1.	In the mediation analysis section, it appears that the authors employed MVMR analysis by using Trait 1 and leukocyte telomere length (LTL) as exposures, while considering Trait 2 as the outcome. This analysis aimed to assess both the direct effect of Trait 1 on Trait 2 and the potential mediated effect of LTL on Trait 2. However, it raises the question of how the mediated effect can be ensured to be independent of other confounding factors, such as Trait 3. Is it possible to conduct MVMR analysis by incorporating additional potential confounders to address this concern?

2. In the mediation analysis section, the authors state, "We tested the mediating role of leukocyte telomere length (LTL) for the relation between 18 non-hematological LTL-modulating traits and lifespan,……". Based on this statement, it can be interpreted that both LTL and the 18 LTL-modulating traits are indeed used as exposures to estimate their individual effects on lifespan. However, considering the results you have presented, it seems the authors used each of the 18 LTL-modulating traits is incorporated individually in a two-exposure MVMR analysis to assess its specific impact on lifespan. To avoid confusion, it could be better the authors make it more clear how MVMR is used in your mediation analysis.

3.	Given the availability of individual-level data for multiple traits, why the authors do not consider an MR analysis using individual-level data for the sex-stratified analysis, rather than solely relying on association analysis.

4.	In the MR analyses, the authors have indeed considered sensitivity analysis using methods like MR-Egger and weighted-mode. However, it is important to acknowledge that these methods are primarily designed to address uncorrelated pleiotropy. Other confounding factors, such as correlated pleiotropy and population stratification, and sample overlap, can also contribute to false positives in MR analysis. It would be valuable if the authors consider sensitivity analysis using methods specifically designed to handle these types of confounding factors, such as CAUSE (PMID: 32451458) and MR-APSS (PMID: 35787050). Additionally, the use of negative controls can be helpful in examining confounding factors.

5.	The authors have revealed several findings in their analysis, for example MR analysis have identified 23 traits that modulating LTL. I think it could be beneficial to consider replication studies using independent datasets to validate these findings.

6. I find the presentation of the results in Figure 2 to be somewhat cluttered and challenging to discern relevant and significant information.

**Authors’ response**

Reviewer 1

This manuscript reports an investigation of the causes and consequences of inter-individual variability in leukocyte telomere length. Using data from the UK Biobank, the authors conduct bidirectional Mendelian randomization analyses, identifying numerous diseases, traits, and biomarkers that show evidence of affecting and/or be affected by LTL. Overall, the work is rigorous and comprehensive, and the paper is well-written. The primary limitation of this paper is the limited novelty. While there are a few novel associations reported here, the majority of the results are confirmatory, having previously been reported, either based on UK biobank data or independent data sets. A few additional concerns are described below.

We thank the reviewer for his/her overall positive assessment. Part of our manuscript (i.e., forward MR analysis) indeed confirms previous findings, which we consider an important part of each scientific project as it ensures that discoveries are built on solid foundations. Yet, our manuscript goes much further than previous work. Notably, we use a much **more comprehensive MR methodology** (i.e., multiple MR methods that account for various potential violations of the assumptions) and estimate both the **reverse effect** of traits on telomere length, as well as the **mediatory role of telomere length**, which to our knowledge has not been studied so far. This is a neglected aspect of telomere epidemiology: while many studies established how telomere shortening correlates with diseases and in some cases established causality, it has not been extensively explored whether telomere shortening is a cause, a consequence, a mediator or a mere correlate of unhealthy aging and diseases. These innovative analyses also shed light on new biology, such as the discussed link between telomeres and female reproductive events such as childbirth and menopause, or illustrate the small, albeit significant role of telomere length in mediating several causal relationships, including those on lifespan. In response to a comment of another reviewer, our mediation analysis additionally revealed that 14.45% of the effect of depression on LTL is mediated through C-reactive protein levels. In our revised manuscript, we aimed at bringing these novel aspects under the spotlight.

1. "we show that LTL partially mediates the impact…" This is worded too strongly, MR assumptions cannot be proved and lifespan is not measured directly.

We toned down this sentence in the abstract:

“*Through multivariable MR, we show that LTL* ***may*** *partially* ***mediate*** *the impact of educational attainment, body mass index, and female age at childbirth on* ***proxied*** *lifespan.*”

2. Why is mother and father's age (at participant birth) analyzed in the "complex trait" section? Should those not be considered predictors of TL and put in the prior section?

In the "*Age and sex are the main predictors of LTL variability*" section, we present the Lasso regression results obtained based on unadjusted LTL, thereby emphasizing the role of age and sex. As stated (lines 43-45):

“*To further explore factors contributing to LTL variability, we included 80 traits (Table S1) with < 7% missingness rates* ***as predictor variables in a Lasso regression model****. Traits retained included age, sex,...*”

Parental ages at participant birth were investigated using LTL adjusted for age, age^2^, genotyping array, sex, and the interaction of the latter with the priors as stated (lines 54-56):

“*Considering the strong correlation between age and sex with LTL, we* ***adjusted LTL for age, age^2^, genotyping array, sex, and the interaction of the latter with the priors*** *and regressed adjusted LTL* ***(hereafter referred as LTL)*** *on 166 traits through linear regression…*”

These covariates were selected based on the results of the Lasso regression. For this reason, these traits are not discussed in the former section, even though we understand that these traits could technically be predictors of LTL (as is the case for many other traits that we investigate in the later sections). Instead, we present these results in the "*LTL broadly associates with complex traits*" section, which discusses observational findings that MR could not examine due to the lack of available GWAS/meaningful genetic instruments, now explicitly stated (lines 64-66):

“*As these traits* ***cannot be genetically instrumented, MR is not applicable.*** *As such, the observational nature of our analysis prevents us from further dissecting the effects of paternal versus maternal age at birth on LTL.*”

To prevent confusion arising from whether results are based on adjusted or unadjusted LTL, we believe it is appropriate to maintain the current organization.

3. Fig 3: in my view the figure is a bit misleading. It is well known that LTL shortening is most rapid during development and early life (when stem cells are dividing rapidly to support growth and development). Also there is no actual data prior to age 40, so the trajectory shown from age 0 to 70 seems to send the wrong message. At least on first look. Also, technically "shortening" should not be used in the title, as you're looking at the association between LTL and age, not shortening among participants. Also the meaning of "predicted LTL" vertical axis label is unclear.

We agree that the original figure could be misleading at first glance. In response, we've modified the figure by starting the x-axis at age 18. This adjustment is informed by the fact that i) estimated trends prior to 40 years is based on extrapolation (along with information on pregnancy/menopausal timing) and ii) previously published data (Aubert et al. 2012; PMID: 22661914) identified a change in the rate of telomere length decline in leukocytes around 18 years of age, increasing the chance that the interpolation does not hold over the range of 0-18 years.

To make it clear that “predicted LTL” corresponds to the regression model fit we changed the figure caption as follows:

“*Figure 3. Schematic representation of LTL shortening across different female reproductive life phases.*

*Relation (β)* ***between predicted (i.e., regression model fit) standardized LTL (y-axis)*** *and age (x-axis) across three female reproductive life periods (red). Dotted vertical lines indicate mean age at first live birth (26 years) and mean age at menopause (50 years). As a comparison, we depict the quadratic LTL regression in males (β_age_; β_age2_; blue). 95% confidence intervals are shown for the predictions. Yellow background indicates the age range for which data are available (40-70 years) and used to build predictions; regions outside this range are extrapolated for males and estimated from age at first live birth and age at menopause information for females. T****he x-axis was set to begin at age 18, reflecting the shift in the rate of LTL decline after puberty [1]. This change cannot be accurately captured by our linear models, which are based on LTL measurements at older ages.***”

Given these specifications, we opted to keep “predicted LTL” on the axis label as this captures the fact that we extrapolate for years before 40.

Finally, the title has been changed to “*Breaking down causes, consequences, and mediating effects of* ***telomere length variation*** *on human health*”.

4. Fig 4. Need to make clear which analyses correspond to women only. I assume anything involving AFB or ALB, what about the rest of the figure?

AFB and ALB are indeed female-specific analyses. To improve clarity, we added an “F” next to female-specific traits in Figure 4, as indicated in the legend:

“***Labels preceded by an uppercase F denote female-specific traits (i.e., age of first and last birth)***”.

5. "As the HBB gene was used as a control for the LTL measurements [20], we removed the single SNP (rs1609812, p = 3.9e−65) associated with this gene (chr11:5'246'696-5'248'301; GRCh37/hg19) to prevent spurious associations." This text needs some additional explanation. And the P-value corresponds to what?

The p-value corresponds to the association strength of the single SNP in *HBB* (rs1609812) with LTL, which was excluded from the IV selection procedure. The reason for that is that *HBB* is used as a control for LTL measurement as described in Codd *et al.* 2021 (PMID: 34611362). To avoid confusion, we have reformulated this sentence and removed this information (lines 394-398):

“*As the HBB gene was used as a control for the LTL measurements,* ***SNPs in this gene (chr11:5'246'696-5'248'301; GRCh37/hg19) that associated with LTL were removed*** *to prevent spurious associations [2].* ***For LTL IVs, this led to the exclusion of a single variant, rs1609812****. Due to the complex long-range linkage disequilibrium (LD) structure of the HLA* ***locus, SNPs found on the extended HLA region*** *(chr6:25'000'000–37'000'000; GRCh37/hg19) were also removed from our IVs [3].*”

6. Seems unlikely to me that TL in leukocytes plays a causal role in non-blood cancers. More likely to be TL in the cancer-relevant tissue, with some SNP effects on TL being shared across tissue types. This could also be true for other diseases, such as lung diseases/traits. This possibility warrants some discussion, as blood cells will not always be the causal tissue/cell type

We agree with this point. Given that telomere length is correlated across tissues (Demanelis *et al.* 2020; PMID: 32913074), we have clarified this limitation in the discussion (lines 278-280):

“***Importantly, the causal relevance of LTL in non-blood-related diseases may be limited, as observed associations could reflect shared genetic effects on telomere length across different tissue types, rather than direct effects of telomere length in leukocytes.***”

7. If your MVMR approach for mediation analysis susceptibility to bias from mediator outcome confounding? Clarify for the reader, as confounders (U) and IV/SNPs for MVMR/mediation analysis are not shown in Figure 1.

In the methods section of the original manuscript, we describe that the mediator is also instrumented:

“*During SNP harmonization, exposure IVs (i.e., trait IVs) were prioritized over* ***mediator IVs*** *(i.e., LTL IVs). Steiger filtering was applied to both exposure IVs with respect to outcome and mediator and to mediator IVs in relation to the outcome.*”

This means we address bias from the mediator to the outcome in the same way we handle bias from exposure to outcome in typical MR analyses. We've added the following explanation to the mediation methods section to make it clearer for the reader (lines 423-433):

“*Excluding hematological traits due to potential confounding, we used a two-exposure* ***multivariable MR (MVMR)*** *framework to individually assess the mediating role of LTL across 18 LTL-affecting traits (p < 0.05/141 = 3.5e−4) on lifespan, proxied through parental lifespan [4]. We further examined the global mediatory role of LTL between each of these 18 LTL-affecting traits and 17 traits causally impacted by LTL (p < 0.05/141 = 3.5e−4). This corresponded to 323 pairs (18 * 18 (i.e., 17 traits + lifespan), excluding one pair as IGF-1 associated with LTL as both exposure and outcome). This sets our significance threshold for the total and indirect effects at p < 0.05/323 = 1.5e−4.* ***IVs for mediation analyses were selected from summary statistics through a two-phase clumping process (see Bidirectional univariable Mendelian randomization) in which harmonized exposure IVs (i.e., trait IVs) were first independently clumped. In the second phase, exposure and mediator IVs (i.e., LTL IVs) were clumped together, prioritizing the former over the latter (i.e., retaining exposure IVs over mediator IVs). Providing MR assumptions hold, by instrumenting both the exposure and mediator we also reduce confounding bias between mediator and outcome.***”

We did not include any confounders in Figure 1 to avoid making the figure too crowded; However, we now specify in the figure caption:

“...*Bottom: Follow-up analyses include exploring the association of female reproductive events with LTL, sensitivity analyses (i.e., implementation of seven complementary MR methods, replication using independent LTL summary statistics from the Vanderbilt University and Marshfield Clinic biobanks [5], controlling for confounding by blood-related traits, and evaluating the MR effect of LTL on sex as a negative control), and perform mediation analysis through multivariable MR.* ***Note that for mediation analyses, both exposure and mediator are instrumented.*** *CHIP = clonal hematopoiesis of indeterminate potential.*”

8. Authors should acknowledge the prior literature on educational attainment at TL in UKB and integrate it into the discussion/interpretation of their analyses involving EA (Amin et al 2022). There are also other prior UKB papers that investigate TL in relation to cardiovascular disease, alcohol, modifiable traits/behaviors, and other phenotypes using MR. These should also be integrated into the discussion.

We deepened our discussion of previous work in our revised manuscript and provide a supplementary table (Table S5) with references to previous works. The discussion now reads as follows (lines 233-258):

“***Others [2, 6, 7] have used MR to estimate the impact of LTL on the human phenome. In contrast to Codd et al. [2], we used summary statistics originating from large consortia, which offer the double advantage of reducing sample overlap between the exposure and outcome sample and have much higher case numbers (and thus power) than in the relatively healthy UKBB. We hypothesize that this explains some of the nine novel findings that are unique to our study, five of which were tested but did not yield significant results (see Table S5). For instance, our results support the Alzheimer’s disease risk-increasing effect of shorter LTL, which was proposed to be driven by promotion of cellular senescence [8, 9, 10, 11, 12, 13]. In line with previous***

***literature [14, 15], our results also suggest that longer LTL increases the risk of systemic lupus erythematosus. Combined with our confirmation that shorter LTL raises rheumatoid arthritis risk, these findings highlight the implication of LTL in autoimmune conditions. Finally, we report a coherent impact of longer LTL on risk for menstrual disorders [6], once again linking LTL to female reproductive health (Figure 3). Overall, this supports the deleterious role of both long and short telomeres in shaping human health.***”

***Unlike previous large-scale studies investigating the link between LTL and the human phenome [2, 7, 5, 16], we also estimated the causal effects of phenotypes on LTL. In line with prior research, alcohol consumption [17], smoking [18, 19], obesity [20], and socio-economic disadvantages [21, 22] emerged as significant contributors to telomere shortening, underscoring the potential benefits of lifestyle modifications. Some of these factors, such as BMI and EA, were found to exert a small, albeit significant proportion of their impact on longevity through LTL. Our findings further indicate that the impacts of both depression and urate levels on LTL are partly exerted through CRP, highlighting inflammation’s role as a mediating factor of LTL shortening [23]. Surprisingly, the positive influence of serum lipid levels on LTL often attenuated the total effect of lipid-trait relationships. These results are unexpected as high cholesterol levels promote oxidative stress [24], which in turn accelerates LTL shortening [25]. Yet they are coherent with recent literature [26] suggesting a nuanced protective role of specific lipids and lipoproteins in maintaining telomere length. However, our inability to replicate these effects when using independent LTL summary statistics, possibly due to lack of statistical power, warrants further investigation into the mechanisms through which elevated lipid levels may support telomere preservation.***”

Reviewer 2

The manuscript by Moix and colleagues explores the causal relationship between leukocyte telomere length (LTL) and various phenotypes using Mendelian randomization (MR). The study employs instrumental variables (IV) derived from summary statistics of a published GWAS study to proxy LTL levels. The investigation into the associations between LTL, a marker of aging, and diverse phenotypes adds an interesting dimension to the field. However, the paper requires substantial revision before it can be considered for acceptance.

We are pleased that the reviewer agrees with the relevance of our approach and we have addressed his/her concerns in the revised manuscript. To address concerns related to the **robustness of MR results**, we implemented MR-APSS, which also considers sample structure and deals with the concerns related to sample overlap and performed an independent replication using LTL summary statistics from Marshfield Clinic’s personalized medicine research project and the biobank of Vanderbilt University Medical Center (Allaire et al. 2023, PMID: 37216007), which have limited overlap with the phenotype summary statistics used. These two approaches confirmed the consistency of our findings. Moreover, we added the limitations of treating diseases as binary exposures in the discussion. We have expanded the methods section and updated the scripts on our GitHub page for improved reproducibility to address concerns related to **clarity**. Finally, we aimed to bring novel biological findings under the spotlight, notably by improving the discussion and adding a table that highlights **which** LTL on trait **findings are novel** (Table S5).

Major Comments:

1. Sample Overlap in Two-Sample MR:

The authors claim to have used two-sample MR methods, but it is noted that all summary statistics are derived from the UK Biobank data. Sample overlap in two-sample MR may introduce bias (Burgess et al. 2016, PMID: 27625185). While Minelli et al. (2021, PMID: 33899104) tested the validity of some common 2-sample methods in a biobank-scale study, but they also indicate that methods like MR Egger are biased. It is also unclear if this extends to the specific methods used in the present study (e.g., MR-PRESSO and MVMR). The authors should either validate their methods for sample overlap or ensure independence between exposure and outcome GWAS samples.

First, we would like to emphasize that we only use MR Egger as a validation approach and do not base any conclusions solely on this method. Sample overlap is often perceived as a source of bias, yet we have previously shown that no overlap may also lead to bias (Mounier et al. 2023 PMID: 37036286) and the extensive simulation studies of our previous paper also showed that the extent of such bias from full sample overlap is minute (<10% relative bias) in the vast majority of simulation settings. We clarified this in the methods (lines 415-421):

“***While sample overlap in two-sample MR may bias results toward observational effects, no overlap may also lead to ’winner’s curse’ bias [27, 28]. Weak instruments can further exacerbate these biases. Although our extensive simulations have demonstrated that these issues lead to mild biases [28], we used MR-APSS [29] (default parameters and LD scores from 1000 Genomes Data), which addresses both sample overlap and pleiotropy. Finally, we replicated the MR analyses using LTL***

***summary statistics generated based on an independent sample (N = 62, 271) from the biobank of Vanderbilt University Medical Center (BioVU) and Marshfield Clinic’s personalized medicine research project (PMRP) [5, 30, 31].***”

Moreover, while it is true that most GWAS were obtained from UK Biobank data, Supplemental Table 6 indicates that for 33 traits (predominantly diseases) we use summary statistics from other sources, that have no or only partial overlap with UKBB.To confirm that the sample overlap is indeed not biasing our results, we ran MR-APSS which accounts for sample structure (Hu *et al.* 2022; PMID: 35787050) and replicated our IVW MR results using LTL GWAS summary statistics from an independent sample (Allaire *et al.* 2023; PMID: 37216007), ensuring that there is no sample overlap between the exposure and outcome summary statistics. Overall, results were highly concordant and we present these new results in the revised manuscript (lines 70-83):

“***To ensure the robustness and reliability of the results, we gauged the reliability of inverse-variance weighted (IVW) significant associations through several approaches (Figure 1). First, we estimated robustness towards MR assumption violation. We applied four additional MR methods implemented in the TwoSampleMR package (MR Egger, simple mode, weighted median, and weighted mode), as well as MR-PRESSO (Figures S3-S4). To mitigate pleiotropy bias, we further implemented a custom approach based on Steiger filtering that requires instrumental variables (IVs) to have a stronger association with the exposure (i.e., LTL) than with any of the other 152 traits analyzed through MR (see Methods; Figure S5). To determine the impact of sample structure, and more notably sample overlap, we ran MR-APSS (which also accounts for pleiotropy) and replicated our IVW results using independent LTL summary statistics from BioVU/PMRP [5]. Estimates were globally consistent across all methods (Figures S3-S4). Regarding replication in BioVU/PMRP, the smaller sample size (N = 62, 271) resulted in larger confidence intervals (CI), yet the correlation between effect sizes remained high (ρ_LTL→T_ = 0.930; ρ_T→LTL_ = 0.874). Specifically, 9 LTL on trait and 5 trait on LTL effects strictly replicated (p < 3.5e−4), while 17 and 12 reached nominal significance, respectively. Only the effect of white blood cell (WBC) counts on LTL had a significantly different effect size (p_diff._ < 0.05/47 = 0.001).***”

***Supplemental Figure 3. MR estimates of LTL on traits across different methods.*** *Mendelian randomization (MR) estimates (x-axis; broken) with 95% confidence intervals (CI) for traits strictly significantly (p < 0.05/141) affected by LTL (y-axis) based on the inverse-variance weighted MR (IVW) effect. Estimates for 7 MR methods are shown: IVW (black), MR Egger (red), MR-PRESSO (green), weighted median (yellow), MR-APSS (cyan), simple mode (grey), and weighted mode (purple). IVW estimates with replication summary ststistics are in blue. Strictly significant effects are shown as full circles; otherwise as empty circles. MR-PRESSO estimates for vessel aneurysms and disorders of menstruation are missing due to the absence of identified outliers. SHBG = sex hormone binding globulin; HDL = high-density lipoprotein; WHR = waist-to-hip ratio; BMI = body mass index; IGF-1 = insulin-like growth factor 1 (see Table S2).*

***Supplemental Figure 4. MR estimates of trait on LTL effects across different methods.*** *Mendelian randomization (MR) estimates (x-axis; broken) with 95% confidence intervals (CI) for traits (y-axis) with strictly significant (p < 0.05/141) inverse-variance weighted MR (IVW) effect on LTL. Estimates for 7 MR methods are shown: IVW (black), MR Egger (red), MR-PRESSO (green), weighted median (yellow), MR-APSS (cyan), simple mode (grey), and weighted mode (purple). IVW estimates with replication summary ststistics are in blue. Strictly significant effects are shown as full*

*circles; otherwise as empty circles. MR-PRESSO are missing for i) light smokers and smoking cessation due to the absence of identified outliers and ii) standing height and mean corpuscular hemoglobin due to convergence issues. IGF-1 = insulin-like growth factor 1; LDL =low-density lipoprotein (see Table S2).*

2. Clarification of Analysis Methods:

It is unclear from the methods section whether mediation analysis and MVMR were performed using individual-level data with one-sample methods or summary statistics with two-sample MR methods. The software or packages used for these analyses need to be specified.

In the methods section of the revised manuscript, we now specify (lines 428-433):

“*IVs for mediation analyses* ***were selected from summary statistics*** *through a two-phase clumping process (see Bidirectional univariable Mendelian randomization) in which harmonized exposure IVs (i.e., trait IVs) were first independently clumped. In the second phase, exposure and mediator IVs (i.e., LTL IVs) were clumped together, prioritizing the former over the latter (i.e., retaining exposure IVs over mediator IVs). Providing MR assumptions hold, by instrumenting both the exposure and mediator we also reduce confounding bias between mediator and outcome.*”

The mediation analysis was not performed with any specific software or packages, but directly and completely performed from base R functions (whose version we now specify in the revised manuscript, i.e., v 4.2.1). This justifies why no packages were listed. Code with our implementation is made available, as mentioned under “*Availability of data and materials”*:

“*Code to reproduce analyses performed in this study is available at https://github.com/cChiiper/UNIL_SGG_MR_LTL.*”

Added “***Software***” section in the revised manuscript (lines 310-312):

“***All analyses were conducted using R version 4.2.1 and Python version 3.11.3. The TwoSampleMR package version 0.5.7 [32] and PLINK v1.90b7 [33] were used. Workflow management was facilitated by Snakemake version 7.25.3 [34].***”

If summary statistics and two-sample MR methods were used (as illustrated by Figure 1), potential biases due to sample overlap need to be addressed. Alternatively, one-sample MR using individual-level data should be considered, with a clear specification of how covariance between the effects of genetic variants on each exposure is treated (Sanderson et al., 2019, PMID: 30535378). Given these uncertainties in the methodology and study design, it is advisable not to draw definitive conclusions from the results until the aforementioned aspects are clarified. The potential for bias in the results needs to be addressed before meaningful interpretations can be made.

Our study is based on summary statistics and two-sample MR methods. As detailed in reply to point 1, we do not think that our results are biased. This was confirmed by a replication study using LTL summary statistics from an independent cohort (Allaire et al. 2023; PMID: 37216007)).

We opted to not run MR with individual-level data as this would result in a lower sample size and loss of statistical power. This is especially true for diseases for which there are not many

cases in the UK Biobank and for which our study benefits from the use of large Consortia summary statistics.

3. Reference 20 and Novelty of Findings:

Reference 20 (Codd et al.) has previously performed one-sample MR between LTL and various traits and diseases (93 quantitative traits and 123 diseases). The authors should clarify if they found any novel associations when considering LTL as an exposure. Emphasizing the novelty of findings, if present, is crucial to distinguish this research from previous studies.

We have now updated the discussion for the parts of the analyses that comprise mostly confirmatory work using a more comprehensive MR approach. Moreover, we now provide a supplementary table that compares our estimates to those of Codd *et al.* and provides further citations for previous MR studies (lines 233-244).

*“****Others [2, 6, 7] have used MR to estimate the impact of LTL on the human phenome. In contrast to Codd et al. [2], we used summary statistics originating from large consortia, which offer the double advantage of reducing sample overlap between the exposure and outcome sample and have much higher case numbers (and thus power) than in the relatively healthy UKBB. We hypothesize that this explains some of the nine novel findings that are unique to our study, five of which were tested but did not yield significant results (see Table S5). For instance, our results support the Alzheimer’s disease risk-increasing effect of shorter LTL, which was proposed to be driven by promotion of cellular senescence [8, 9, 10, 11, 12, 13]. In line with previous literature [14, 15], our results also suggest that longer LTL increases the risk of systemic lupus erythematosus. Combined with our confirmation that shorter LTL raises rheumatoid arthritis risk, these findings highlight the implication of LTL in autoimmune conditions. Finally, we report a coherent impact of longer LTL on risk for menstrual disorders [6], once again linking LTL to female reproductive health (Figure 3). Overall, this supports the deleterious role of both long and short telomeres in shaping human health.***”

To better distinguish our work from previous work, we later in the discussion state (lines 260-261):

“***One of the most intriguing findings of our study is the causal relationship between delayed AFB and ALB and extended LTL in females, which was only partially confounded by SES.***”

We also provide an in-depth follow-up analysis linking female reproductive timing with LTL.

4. Power and Weak Instrument Bias:

The sample sizes of cases for many diseases are low, potentially resulting in a low percentage of variance explained by instrumental variables. F-statistics for all IVs for each phenotype in univariable MR should be provided to demonstrate that the MR is not suffering from weak instrument bias (not just conditional F-stat in MVMR). The authors should discuss the power of MR analyses given the current sample size.

When selecting instruments based on a genome-wide significance threshold (p < 5e-8), an approximation using the standard normal distribution results in a t-value of 5.45. When computing the F-statistic for a single SNP (k = 1), the F-statistic is closely approximated by the square of the t-statistic. Therefore, even adopting a more conservative approach, we can anticipate that all individual F-statistics will surpass at least 20. Conventionally, a threshold of 10 is considered adequate for denoting instrumental variable strength, making it unnecessary to add this information. Moreover, it's noteworthy that the sample sizes for the 52 diseases with available GWAS summary statistics are not that low, given that we aimed at using the largest available Consortia summary statistics. On average there are 34,905 cases, with a median of 17,288 cases. Only systemic lupus erythematosus summary statistics were based on less than 1,000 cases (see Supplemental Table 6). As for an in-depth discussion on the power of MR analyses, we consider it beyond the scope of this paper. Interested readers may refer to the supplemental tables for additional information and are encouraged to use tools such as https://shiny.cnsgenomics.com/mRnd/ for further analysis.

5. Bidirectional MR and Violation of Assumptions:

In bidirectional MR, treating diseases as binary exposures may violate the exclusion restriction assumption. The authors should evaluate and discuss the validity of the method and the interpretation of results in light of this potential limitation (Burgess et al., 2018, PMID: 30039250).

We thank the reviewer for pointing this out. This is indeed a limitation of our study, which we now discuss (296-298):

“***Fifth, treating diseases as binary exposures may violate the exclusion restriction assumption [35], prompting careful interpretation of the effects of hypertension, lipid disorders, and depression on LTL.***”

Minor comments:

1. MR Egger Intercept:

The manuscript should include the intercept of MR Egger regression, not just MR estimates. This provides an indication of pleiotropy when non-null.

We have added the intercept, as well its standard error and p-value for MR Egger in the supplementary Table 2 of the revised manuscript.

2. Exclusion of SNPs in HLA Locus:

The removal of SNPs in the HLA locus from instrumental variables is mentioned in the method section. However, the authors should consider that these SNPs could be significantly associated with certain traits or diseases (e.g., rheumatoid arthritis) and their exclusion may compromise the power of MR.

We understand this concern; however, the exclusion was deliberate due to the HLA region's high polymorphism and extensive linkage disequilibrium, which complicates the interpretation of causal relationships in Mendelian randomization analyses. This practice aligns with common strategies in the field aimed at minimizing bias from pleiotropy and ensuring clearer, more interpretable results. While this may reduce the power to detect HLA-mediated associations, it enhances the reliability of our findings and, in a worst-case scenario, would potentially lead to false negatives.

Nevertheless, we examined the effect of LTL on rheumatoid arthritis, a disease with a strong immune component and a significant MR effect, with and without including HLA SNPs in our analysis: without HLA SNPs (nsnp = 328, b_ivw_ = -0.087, p_ivw_ = 6.13e-05) and with HLA SNPs (nsnp = 349, b_ivw_ = -0.095, p_ivw_ = 4.49e-05). This comparison underscores that including HLA SNPs does not markedly change the effect estimates, indicating that the exclusion of these SNPs does not strongly impact our results.

In summary, addressing these major comments is crucial for ensuring the robustness and validity of the study's findings. Substantial revisions are recommended before the manuscript can be considered for acceptance.

Reviewer 3

Moix et al use Mendelian Randomisation to investigate the associations, causes, consequences of variation in measured leukocyte telomere length using UK Biobank data. Their findings will be of interest to those studying telomere biology, ageing and age-associated diseases. The statistical approaches and choice of covariates appear appropriate. Interpretation of results is mostly appropriate, with some exceptions. Below I have some suggestions aiming to further improve the manuscript.

We thank the reviewer for the overall positive feedback and some of the suggestions that have brought interesting new insights/discussion points to the manuscript, notably those related to the measurement of telomere length from blood samples and impact of clonal hematopoiesis. While including observational analyses of clonal hematopoiesis falls outside the scope of our study, we have enriched our manuscript with MR-based analyses assessing the implications of clonal hematopoiesis on our results. We conducted an additional MVMR analysis that includes relevant blood traits as covariates to ensure that the latter do not bias our results. These added analyses corroborate the robustness of our original findings, strengthening their validity.

Major

1. The authors need to consider more carefully that leukocyte telomere length (LTL) is a measurement of telomere length of a diverse mixture of leukocytes and can be affected by somatic mutations: In particular, the phenomenon of clonal hematopoiesis has been variously found to be associated with non-hematological diseases, including cardiovascular disease. Clonal hematopoiesis is also associated with greater attrition of telomere length and this may be the basis for the association between lower LTL and hematological cancer (as clonal hematopoiesis is the precursor to many blood cancers). The authors need to consider the above and ensure their analysis takes it into account. They should at least repeat their analyses after excluding participants with clonal hematopoiesis. It may be also informative to examine the interaction of clonal hematopoiesis and LTL in driving diseases, but admittedly this will not be straightforward.

This comment brings up an interesting point. While earlier research suggests that there is a negative link between clonal hematopoiesis of indeterminate potential (CHIP) and telomere length, MR studies (e.g., PMID: 35385311; PMID: 37140166), indicate that the genetic effect of telomere length on CHIP might be stronger than the reverse effect. To explore this, we used the latest CHIP GWAS summary statistics (PMID: 36450978) and found a significant causal influence of LTL on CHIP, but not the reverse. This suggests that while CHIP may play a role in some of the assessed diseases, it likely doesn't bias the MR findings. We added these results in the revised manuscript under the new “***Sensitivity analyses***” section as follows (lines 85-99):

“*Second, we sought to assess if our results could be confounded by hematological factors, given that we use telomere length assessed in leukocytes. We therefore adjusted LTL for eosinophil, lymphocyte, monocyte, neutrophil, platelet, red blood cell, reticulocyte, and WBC counts in addition to core covariates. Regressing this new variable on the same 158 traits (i.e., 166 traits, excluding the 8 blood count traits we corrected for), we obtained highly similar effect sizes (ρ = 0.98). Only associations with smoking status (p_diff._ = 1.4e−9),*

*smoking cessation (p_diff._ = 5.7e−6), as well as mean corpuscular hemoglobin (MCH; p_diff._ = 3.3e−26) were significantly reduced (p_diff._ < 3.5e−4), yet remained significant. Association with total bilirubin (p_diff._ = 1.4e−5) was lost, while the one with phosphate levels (p_diff._ = 8.0e−5) became significant (Figure S6).* ***As a relationship between LTL and clonal hematopoiesis of indeterminate potential (CHIP) has been suggested [36, 37], we conducted bidirectional MR analysis to assess whether CHIP [38] could confound LTL associations. While long LTL had a causal impact on CHIP incidence (α_LTL→T_ = 0.147; p = 3.0e−9), we did not identify a reverse effect (α_T→LTL_ = 0.040; p = 0.618). This suggests that confounding of our MR analyses by CHIP is an unlikely scenario. MVMR analysis with blood counts (with significant association with LTL), MCH, CHIP, and LTL as exposures against LTL-impacted traits also did not reveal significant effect changes in effect sizes, confirming that neither CHIP nor other hematological parameters biased our results (see Table S3).***”

As an additional precaution, we assessed whether any of the LTL instrumental variables were located close to (± 100 kb from gene) any of the 23 CHIP genes identified by Kessler *et al.* 2022 (PMID: 36450978). Only one variant, rs137856292 located 81’685 bases from *PRPF8*, would be excluded. Given the large number of LTL instrumental variables (N_min_ = 185 and N_max_ = 335), a single variant is unlikely to strongly impact results.

As our manuscript is centered on studying telomere biology through an MR framework, redoing all observational analysis by excluding participants with clonal hematopoiesis falls out of the scope of this study, especially considering the time it would take to get access to the UKBB CHIP data, which we currently do not have access to. Furthermore, such stratified analysis would likely lead to collider bias, given the dominant LTL-to-CHIP effect.

Minor

1. Table 1: as this is a MR study, it would be better to avoid using the term "determinants" of LTL for these observational analyses and use terms like "associations" (saving terms that imply causality for MR analyses)

We agree with the reviewer; Table 1 title has been changed to: “*Major* ***associations*** *with LTL*”. The text (line 54) was also changed from “*Age and sex being the major determinants of LTL…*” to “***Considering the strong correlation between age and sex with LTL****…*”

2. The reasons for the association of paternal age with LTL may be very different to those for maternal age. As paternal and maternal age correlate between them, it would be interesting to understand the degree of independence of the association of LTL with paternal vs maternal age (e.g. by conditioning one or other).

Acknowledging the importance of the question raised, we assessed the association with a multivariable model, as illustrated in Supplemental Figure 2, finding a slightly greater reduction of the maternal vs paternal age effect on LTL. Yet, the significant correlation between maternal and paternal ages complicates the distinction of their individual impacts on LTL. Despite literature suggesting a positive relationship between paternal age and sperm telomere length (PMID: 31138065), our study's observational nature and the inapplicability of Mendelian randomization (as these traits can’t be genetically instrumented)

prevent definitive isolation of the effects of paternal versus maternal age on LTL. To clarify this limitation, we added the following sentence:

“***As these traits cannot be genetically instrumented, MR is not applicable. As such, the observational nature of our analysis prevents us from further isolation of the effects of paternal versus maternal age at birth on LTL.*** ”

3. It is recognized that lymphocyte telomere length differs from neutrophil LTL. In this light, the observed associations of LTL with lymphocyte, white blood cell, monocyte, eosinophil and neutrophil counts may simply reflect these differences in telomere length between myeloid and lymphoid cells, rather than a "causal" consequence of telomere length. This should be discussed. Also including these blood counts as covariates may change some of the results pertaining to other diseases, and should be attempted.

The original manuscript (Supplemental Figure 8, now Supplemental Figure 6) included blood counts as covariates in observational correlation. This analysis only found a significant difference in effects size for 6 traits upon adjustment for hematological traits. To complement this, the revised manuscript additionally contains MVMR analyses with blood counts, mean corpuscular hemoglobin (which also showed significant MR effects with LTL), clonal hematopoisis, and LTL as exposures and LTL-affected traits as outcomes. Results are described as stated earlier in the “***Sensitivity analyses***” section (lines 96-99):

“***MVMR analysis with blood counts (with significant association with LTL), MCH, CHIP, and LTL as exposures against LTL-impacted traits also did not reveal significant effect changes in effect sizes, confirming that neither CHIP nor other hematological parameters biased our results (see Table S3).***”

We also mentioned this point under the limitations of the study (lines 278-282):

“***Importantly, the causal relevance of LTL in non-blood-related diseases may be limited, as observed associations could reflect shared genetic effects on telomere length across different tissue types, rather than direct effects of telomere length in leukocytes. Furthermore, we cannot exclude that since telomere length was measured in leukocytes, it made finding associations with hematological traits more likely, despite our sensitivity analyses showing robustness against confounding by hematological factors.***”

4. Line 218: the discussion about depression causing oxidative stress may be a bit simplistic, particularly as it ignores known links between depression and other factors that affect LTL including many identified within this manuscript (e.g. physical activity, diet, smoking, alcohol intake, WHR, social habits etc)

This is a fair point. Ideally, one could disentangle this through MVMR. While it is difficult to instrument most lifestyle factors of potential relevance, we can at least assess if part of the effect of depression is mediated through CRP. Findings showed that 14.45% of the effect of depression on LTL is mediated by CRP. In light of these new results, we revised the manuscript to include the fact that depression may indirectly affect LTL through such factors, while still emphasizing that this only represents one possible explanation:

“***We speculate that depression could accelerate LTL shortening by influencing lifestyle factors that promote oxidative stress and inflammation, both critical modulators of LTL [39, 40, 41].*** *The latter is supported by a negative causal effect of the inflammation marker C-reactive Protein (CRP) on LTL (α_T→LTL_ = −0.037; p = 9.3e−10).* ***While it is challenging to genetically instrument lifestyle factors, we conducted mediation analyses to explore the extent to which smoking cessation, frequency of alcohol intake, body mass index (BMI), and CRP levels mediate the effect of depression on LTL. We found that only CRP significantly mediated part of the relationship between depression and LTL (P_M_ = 14.45%; 95%-CI[3.31%; 32.1%]).***”

5. Line 233 onwards: consider changing "birth" to "childbirth", to avoid readers thinking that the former refers to the participants' birth or factors related to it (e.g. prematurity, birth weight etc). Also, "Figure 3" should say "Age at first (child)birth" not "Age of first birth".

We revised the manuscript using "age at first live birth" as the trait is defined in the UK Biobank, or simply "childbirth" when the context permitted. Figure labels have been updated accordingly.

***Figure 3. Schematic representation of LTL shortening across different female reproductive life phases.***

*Relation (β) between predicted (i.e., regression model fit) standardized LTL (y-axis) and age (x-axis) across three female reproductive life periods (red). Dotted vertical lines indicate mean age at first live birth (26 years) and mean age at menopause (50 years). As a comparison, we depict the quadratic LTL regression in males (β_age_; β_age2_ ; blue). 95% confidence intervals are shown for the predictions. Yellow background indicates the age range for which data are available (40-70 years) and used to build predictions; regions outside this range are extrapolated for males and estimated from age at first live birth and age at menopause information for females. The x-axis was set to begin at age 18, reflecting the shift in the rate of LTL decline after puberty [1]. This change cannot be accurately captured by our linear models, which are based on LTL measurements at older ages.*

6. In relation to telomere attrition in women and childbirth/menopause, it is not clear if the authors had information about the age at menarche. If they do, it may be helpful to examine its relevance, as women with early menarche were reported to have children slightly earlier than those with later menarche in some studies.

We had information about menarche, however this trait never showed any significant association with LTL. Reproductive lifespan (age at menopause - age at menarche) showed an associative link, but likely driven by the association observed by age at menopause, which was also significant by itself. One possible explanation – besides that there is truly no relation between these two traits – is that age at menarche is likely to be reported with much less accuracy than menopause, given that the event took place several decades ago. Furthermore, prior research (Aubert et al. 2012; PMID: 22661914) has shown a shift in the pace of telomere shortening in leukocytes around the age of 18. Given that menarche occurs before this age, our study may not accurately assess LTL-menarche relationship due to the lack of LTL data around this age period. Therefore, we've updated the results and discussion sections of our manuscript to acknowledge this limitation. We now added the following sentence in the “*Female reproductive traits*” section (line 137):

“***Testing for age at menarche showed no association with LTL (β = 0.004; p = 0.113).***” The discussion now includes the following paragraph (lines 267-268):

“***Notably, no significant association between age at menarche and LTL was found. While this could reflect a genuine absence of association, inaccurate reporting of age at menarche and inability to assess shifts in telomere shortening rate before adulthood [1] could also contribute to this negative result.***”

7. Under "renal health" the authors implies that the association of Urate and LTL implies a link between LTL and renal function, but urate is not a great measurement of the latter (in line with this, much of the effect of urate on LTL is linked to high CRP).

We apologize for the misunderstanding. We agree with the reviewer that cystatin C is the best measurement of renal function and the link between LTL and the latter was mainly based on the fact that cystatin C was retained among the top LTL predictors. Yet, we did not identify any significant MR association between cystatin C and LTL. Because urate can under certain circumstances be a biomarker of renal health, we include the discussion on urate findings in that section, even though we are aware that unlike cystatin C, it is not specific to kidney health and can be a biomarker of many other conditions. We now realize that this can be misinterpreted.

Cystatin C is a good measure if renal function, but can also be affected by inflammation. It would be good to comment on the impact of CRP on the cystatin-LTL association and also on any link between creatinine and LTL (creatinine is a less good measure of renal function than cystatin when there is no inflammation, but probably a better one when there is).

As described above, we observe a significant observational correlation between LTL and cystatin C but we do not observe any MR effect. Similarly, serum creatinine shows a negative correlation (*β* = -0.010, p = 3.5e-8) with no significant MR effects (see Figure 2).

If creatinine is not associated with LTL, then it may be unsafe to link LTL with renal function.

Although creatinine is associated with LTL, the absence of MR effects led us to refrain from making claims about LTL and renal functions. Consequently, we have renamed the “*Renal health section*” to “***Urate***”, which now reads as follows (lines 160-164):

“***Urate levels, also retained as a relevant predictor of LTL by the Lasso regression analysis, displayed a negative association with LTL (β = −0.025; p = 2.1e−44). As previously reported [42], MR analyses showed that elevated urate levels decreased LTL (α_T→LTL_ = −0.042; p = 9.4e−18), possibly due to increased cellular stress and reactive oxygen species production [43] (Figure 2f). The urate-LTL association was significantly mediated by CRP, confirming the role of inflammation in this process (P_M_ = 34.7%; 95%-CI[17.1%; 55.6%]; see Table S4).***”

Note that we kept urate under “Renal” category in Figure 2, as we did not find any better suited category but thanks to the textual changes, it should now be clear to the reader that we do not imply that the urate-LTL link necessarily relates to renal function.

8. Under "hepatic biomarkers" the authors should consider that hepatic disease is a feature of telomere biology disorders, as is pulmonary fibrosis, and add some relevant discussion.

In the updated manuscript we now discuss this matter. As MR was not performed for cirrhosis we now performed an additional analysis using the precursor nonalcoholic fatty liver disease as a proxy in order to get a larger sample size. The section has now been updated with the following (lines 179-184):

“***Hepatic disorders, which can lead to altered levels of AST [44], are a feature of telomere biology disorders [45]. Accordingly, we observe an association between LTL and liver fibrosis/cirrhosis (β = −0.012; p = 1.2e−10). However, nonalcoholic fatty liver disease, which reflects the early stages of liver disease and has GWASs with larger sample sizes, does not demonstrate any significant MR effects (p = 0.212). Nevertheless, these results underscore the potential role of telomere-driven cellular aging in hepatic function and/or inflammatory processes.****”*

9. The authors should also discuss the fact that LTL is a measure of blood cell telomeres and whilst this correlates with telomere length in other tissues, this correlation is not perfect. This is relevant to the fact that the tissue-specificity of telomere biology disorders (where the telomere length of individual tissues may be a stronger determinant of phenotypes) is not the same as the diseases linked to LTL.

We complemented the limitations of the study as follows (lines 276-282):

“*Second, although LTL and telomere length in other tissues are correlated [46], this proxy might miss more subtle and tissue-specific relations between telomere length and the phenome.* ***Importantly, the causal relevance of LTL in non-blood-related diseases may be limited, as observed associations could reflect shared genetic effects on telomere length across different tissue types, rather than direct effects of telomere length in leukocytes. Furthermore, we cannot exclude that since telomere length was measured in leukocytes, it made finding associations with hematological traits more likely,***

***despite our sensitivity analyses showing robustness against confounding by hematological factors.***”

Reviewer 4

Review of GBIO-D-23-01573 Moix et al., "Breaking down causes, consequences, and mediating effects of age-related telomere shortening on human health"

Overall, this is a strong paper using Mendelian randomization to examine the role of leukocyte telomere length (LTL) on a variety of serious health problems that potentially affect lifespan. It uses the UK biobank data on telomere length of almost a third of a million research participants. This large dataset offers substantial statistical power to detect relatively small effects between genomic instrumental variables and health outcomes that are potentially mediated via LTL. Most of the comments I have are minor corrections or requests for more details or clarification.

We appreciate the positive assessments and detailed comments that allowed us to re-focus and improve the clarity of our manuscript.

An important aspect of this paper is that it explicitly considers bidirectional causation between LTL and health outcomes. Thus, the authors identify 23 significant causal effects of traits on LTL and 26 significant effects of LTL on traits. These estimates have been obtained by fitting two unidirectional models, one in each causal direction. While these are unlikely to be distant from those that would be obtained from an explicit bi-directional causal model. Several such models have been described in the literature, so it seems reasonable that they should be employed in the present case.

The only traits with potential bias will be traits for which we find a bidirectional effect with our unidirectional approach. This concerns lymphocyte count, mean corpuscular hemoglobin, eosinophil count, red blood cell count, and IGF-1, as we have now highlighted in purple on Figure 2. Such bidirectional causal effects introduce an infinite causal loop, which lead to a discrepancy between the total causal effects and the direct causal effects.

_1− α* × β*_β =^β*^

α*

The potential bias can be obtained from: α = and where 1− α* × β*

^*^ = �������� �������������� ������������

α = ������������������ ���������� �������������� ������������ ; α ; ^*^ = �������� �������������� ������������

β = ������������������ ���������� �������������� ������������ ; β . Taking the product of the _1−α*β*_ =^2αβ^

1

two equations and solving it for α *× β * yields that ~~.~~ This multiplier is 1+4αβ−1

close to 1 when the product of the estimated total causal effects is small. The table below shows the values we observed for^2αβ^

1+4αβ−1

| **Trait** | **Forward**  **Reverse**  2αβ  **LTL → Trait (ivw)**  **Trait → LTL (ivw)**  1+4αβ−1 |
| --- | --- |
| **Lymphocyte count MCH**  **Eosinophil count** | -0.06610020 -0.04491192 1.002960 -0.19513350 -0.03420895 1.006631 -0.10496616 -0.04246606 1.004438 |
| **Red blood cell count** | 0.14038262 0.02889352 1.004040 |
| **IGF-1** | 0.08914635 0.02504832 1.002228 |

Thus, the direct causal effects for these five traits might be slightly overestimated, but this small potential bias can be considered negligible, especially as, to our knowledge, there is no relevant MR explicit bi-directional model that would be suited or applicable. Two methods that we consider relevant are LHC-MR (PMID: 34907193) and MR-DoC2 (PMID: 36322200). LHC-MR primarily addresses heritable confounders, which falls outside our main area of focus. Implementing MR-DoC2 necessitates the use of individual-level data and details from relatives. Without any anticipated substantial benefits, we have decided against implementing it, yet we now address the matter in the discussion (lines 294-296):

“***Moreover, we derive our causal estimates from two unidirectional models, each fitting a different causal direction. Not using an explicit bidirectional model may marginally overestimate the effect sizes for the five hematological traits with bidirectional MR effects, but the expected bias is minimal [47].***”

In common with many such investigations, the Type I error rate was not corrected for once the sample was subsetted into males and females. To mitigate this, the Bonferroni approach is pretty conservative, so it's probably not a big deal, but should be addressed by the authors.

Because of the lack of sex-specific summary statistics, the original manuscript did not contain sex-specific MR results. As we decided to refocus the revised manuscript more strongly on causal links between LTL and complex traits (e.g., see the many sensitivity/replication analyses performed in response to comments from other reviewers), we decided to omit the sex-specific results, which were based solely on observational correlation.

Details:

p.6 line ~53:

Setting non-parous and postmenopausal women's scores to zero for number of years is an unusual choice, since these data are effectively missing, and should not be scored as zero. It would help to revise this coding and report the effect of so doing on the parameter estimates. Hopefully, not much will change, but estimates and their standard errors seem likely to be less biased by fitting a truly bidirectional structural equation model (SEM).

One important consideration is that the score of non-parous and postmenopausal women is set to 0 only for a specific time period. The three phases are outlined by time periods defined by first live birth and menopause. Therefore, data on time spent in these periods is inherently not missing for women who, for example, never gave birth, as they did not experience the post-birth and pre-menopause period (phase 2). Consequently, we consider assigning a duration of 0 years for phase 2 to these individuals a logical approach. The same logic applies for females who have not undergone menopause yet and have thus not spent any time in phase 3. Note that females with genuinely missing or inconsistent data were excluded from the analysis, as was specified in the methods section of the original manuscript:

“*Female participants who had their first child post-menopause, lacked a menopausal status (#2724) or age at menopause (#3581), or did not specify childbirth events (#2734) or age at first childbirth (#2754) were excluded from this analysis.*”

p.6 line 98:

Some fairly large proportions of the sample were lost due to regular regression's weakness that no missing observations should be present for the independent variables. This issue would be better handled via SEM, for which likelihoods can be calculated for participants with partially missing data. If this approach is not followed, then at least the means and variances of participants who were excluded should be presented alongside those who were not. Doing so will enable checking to see if the missing data points are missing completely at random, or if some other missingness mechanism is at play whose effects should be controlled.

We attempted imputation methods to address data missingness utilizing R libraries like mice or VIM, but ultimately decided to not include these results in our manuscript. This decision was informed by the substantial computational demands and marginal expected benefits of these methods. Additionally, we considered simpler imputation strategies, such as using mean and variance, but deemed them not sufficiently useful (and leading to regression dilution bias) for addressing the complexities of the data. Upon reflection, we concluded that data imputation, within this specific context, does not provide a meaningful advantage and, therefore, does not justify the extensive resources required for its implementation.

Instead, we focused our analysis on traits that were consistently present across a majority of participants. Regardless of the missingness threshold applied, the analysis consistently highlighted sex and age as the primary contributors to variance, i) justifying adjusting for these two variables in downstream analyses and ii) underscoring the phenome's limited predictive capacity concerning LTL variability. Furthermore, the examination of whether data points missing from the UK Biobank are missing completely at random constitutes a distinct and complex issue, and therefore, falls beyond the scope of this study. Nevertheless, we examined differences in LTL between participants with and without missing data, finding that missingness, similar to many health traits, is correlated with LTL. We thus complemented the limitations of the study as follows (lines 286-288):

“***Third, Lasso regression in our study was limited to data without missing values. Comparisons between cases with and without missing data showed shorter LTL means in the former, indicating that missingness, previously correlated with several health traits [48], constitutes an additional limitation to our findings***.”

p.6 line 100:

How were the effects of array treated? Two methods spring to mind: dummy coding yes or no for each array that was used, to end up with as many covariates as there were arrays, minus one. Another would be to estimate their influence as a random effect. It is important to clarify these details to give others even a slight chance of reproducing the results.

The sex-stratified analysis section has now been removed, as explained in the reply to a previous point of the reviewer. The point remains relevant, as we also corrected for genotyping array in other analyses. This information was now added under the “*Single trait linear regression*” section (lines 362-363):

“***We adjusted LTL by regressing out age, age^2^, genotyping array, sex, and the interaction of the latter with the priors as fixed effects****…*”

p.7 line 126:

Why were the additional IVs removed? Some greater clarity is needed with the steps that eliminate data; doing so can greatly affect results and reproducibility.

IVs that are not shared across exposure/outcome summary statistics (and thus not usable) or those that could violate the MR assumptions are often excluded as standard practice in MR. First, SNPs are removed during harmonization between exposure and outcome datasets; SNPs not present in the outcome data are excluded. Subsequently, SNPs are subject to two filtering steps specific to each exposure-outcome pair: the first ensures similarity in allele frequencies between exposure and outcome (as large discrepancies could indicate population differences or erroneous data), and the second verifies that the effect of the exposure on the outcome is larger than the reverse effect (to avoid reverse causality). SNPs in the *HBB* gene were removed to prevent false associations due to its role as a control in LTL measurements, and SNPs in the HLA region were excluded due to their complex linkage disequilibrium pattern and extreme pleiotropy (common practice in the field). Note that we examined the effect of LTL on rheumatoid arthritis, a disease with a strong immune component and a significant MR effect, with and without including HLA SNPs in our analysis: without HLA SNPs (nsnp = 328, b_ivw_ = -0.087, p_ivw_ = 6.13e-05) and with HLA SNPs (nsnp = 349, b_ivw_ = -0.095, p_ivw_ = 4.49e-05). This comparison underscores that including HLA SNPs does not markedly change the effect estimates. The concerning method section has now been updated as follows to improve clarity:

“***Harmonized SNPs significantly associated (p < 5e−8) with the exposure were clumped (p1 = 0.0001, p2 = 0.01, kb = 250, and r2 = 0.01) with PLINK v1.9 [33] and retained as instrumental variables (IVs). As the HBB gene was used as a control for the LTL measurements, SNPs in this gene (chr11:5’246’696-5’248’301;394 GRCh37/hg19) that associated with LTL were removed to prevent spurious associations [2]. For LTL IVs, this led to the exclusion of a single variant, rs1609812. Due to the complex long-range linkage disequilibrium (LD) structure of the HLA locus, SNPs mapping to that region (chr6:25’000’000–37’000’000; GRCh37/hg19) were also excluded from our IVs [3]. For each exposure-outcome pair, further IVs were removed based on difference in allele frequency (≥ 0.05) and Steiger filtering (Z ≤ −1.96).*”**

p.7 line 137:

Similar to prior comment, "we first 137 filtered genome-wide significant exposure SNPs and harmonized these SNPs across all 152 traits" is not clear how the harmonization was done.

Through our reply to the previous comment and adding the following details (now with one added GWAS trait), we hope to have clarified how harmonization was achieved (lines 408-411):

“*...we first filtered genome-wide significant exposure SNPs and harmonized these SNPs across all* ***153*** *traits with available GWAS summary statistics, i.e., v****erified that SNPs are present across the 152 traits + LTL summary statistics. This step is carried out before clumping to guarantee that the identified IVs are consistently present across all outcomes, enabling subsequent comparisons.***”

p.16 top:

"Short LTL correlated with increased risk for cardiovascular and pulmonary conditions, reflecting previous findings

289 [12, 48]." This is an accurate statement, but readability is adversely affected because the descriptor "short" reverses the sign of the associations reported in the following sentences. I think it would be better to state it the other way around, that long telomeres are protective, having less adverse outcomes, then the signs of the coefficients agree with the statement.

The sentence has been reformulated so that the signs of the coefficients align with the statement (line 186):

“***Longer*** *LTL correlated with* ***decreased*** *risk for cardiovascular and pulmonary conditions, reflecting previous findings…*” so that the signs of the coefficients align with the statement.

p.21 line 361:

The word "variation" is the wrong one for this context. It isn't that variation in LTL increases risk, it's the value of an individual's LTL 'score' that does so. Variation might mean within person or across persons, and in neither case is it consistent with what is reported in the paper, i.e., one score per person.

This point has been taken into consideration. Due to other changes, the updated manuscript no longer contains that sentence.

p.22 line 378:

is should be are; data are plural.

This has been corrected throughout the manuscript.

p.22 line 388:

It would strengthen the paper to note whether parity per se is associated with longevity. In general, the discussion seems to be few connections to the prior literature, or even that which was discussed in the introduction.

As stated in the original manuscript, we find that age at childbirth associates with longevity:

“...*Results emphasized the partial mediating role of LTL and EA on the effect of AFB on lifespan.*”

The following was added to the discussion, as parity was also previously reported to be associated with longevity (lines 269-271):

“***Extending prior literature [49], our findings suggest that delayed female age at childbirth increases longevity partly through telomere length, even though we cannot exclude partial confounding by SES, which also significantly influences this relationship.***”

Additionally, we have significantly enhanced our discussion by incorporating a more comprehensive review of existing literature (lines 233-258):

“***Others [2, 6, 7] have used MR to estimate the impact of LTL on the human phenome. In contrast to Codd et al. [2], we used summary statistics originating from large consortia, which offer the double advantage of reducing sample overlap between the exposure and outcome sample and have much higher case numbers (and thus power) than in the relatively healthy UKBB. We hypothesize that this explains some of the nine novel findings that are unique to our study, five of which were tested but did not yield significant results (see Table S5). For instance, our results support the Alzheimer’s disease risk-increasing effect of shorter LTL, which was proposed to be driven by promotion of cellular senescence [8, 9, 10, 11, 12, 13]. In line with previous literature [14, 15], our results also suggest that longer LTL increases the risk of systemic lupus erythematosus. Combined with our confirmation that shorter LTL raises rheumatoid arthritis risk, these findings highlight the implication of LTL in autoimmune conditions. Finally, we report a coherent impact of longer LTL on risk for menstrual disorders [6], once again linking LTL to female reproductive health (Figure 3). Overall, this supports the deleterious role of both long and short telomeres in shaping human health.***

***Unlike previous large-scale studies investigating the link between LTL and the human phenome [2, 7, 5, 16], we also estimated the causal effects of phenotypes on LTL. In line with prior research, alcohol consumption [17], smoking [18, 19], obesity [20], and socio-economic disadvantages [21, 22] emerged as significant contributors to telomere shortening, underscoring the potential benefits of lifestyle modifications. Some of these factors, such as BMI and EA, were found to exert a small, albeit significant proportion of their impact on longevity through LTL. Our findings further indicate that the impacts of both depression and urate levels on LTL are partly exerted through CRP, highlighting inflammation’s role as a mediating factor of LTL shortening [23]. Surprisingly, the positive influence of serum lipid levels on LTL often attenuated the total effect of lipid-trait relationships. These results are unexpected as high cholesterol levels promote oxidative stress [24], which in turn accelerates LTL shortening [25]. Yet they are coherent with recent literature [26] suggesting a nuanced protective role of specific lipids and lipoproteins in maintaining telomere length. However, our inability to replicate these effects when using independent LTL summary statistics, possibly due to lack of statistical power, warrants further investigation into the mechanisms through which elevated lipid levels may support telomere preservation.***”

p.22 line 394: "Furthermore, we cannot exclude that the tissue in which telomere length was measured (i.e., leukocytes) biases the observed associations with hematological traits, even if accounting for blood cell type traits did not seem to affect association estimates between LTL and other traits." I think the sentence would work better rewritten as follows: "Furthermore, we cannot exclude that since telomere length was measured in leukocytes, it made finding associations with hematological traits more likely. This inequality between traits may exist even if accounting for blood cell type traits did not seem to affect association estimates between LTL and other traits."

The sentence has been revised as proposed (lines 280-282).

p.23: line 409:

"exogenous and endogenous environmental factors" the distinction between these types of environmental factor first appears here in the conclusion. Either define them here, or define them earlier, but interested readers are likely to want to know which are which.

The sentence was updated as follows (lines 301-303):

“*In conclusion, through usage of rigorous univariable and multivariable bidirectional Mendelian randomization, we identify a complex network of causal relations* ***wherein both exogenous (i.e., lifestyle or environmental) and endogenous (i.e., physiological) factors modulate LTL****…”*

Supplemental Figure 7:

"a) socio-economic status or b) cholesterol medication" in the caption does not agree with the figure itself, in which a) and b) are reversed.

The labels on the figure have been rectified.

***Supplemental Figure 7. LTL associations adjusted for potential confounding variables.*** *Linear regression estimates (x-axis) with 95% confidence intervals (CI) of a) female reproductive traits or b) serum lipid levels (y-axis, left) on LTL, adjusted (pink) or not (black) for potential confounders (y-axis, right), i.e., a) socio-economic status or b) cholesterol medication. Significantly different effects p_diff._ < 0.05 are shown as circles, otherwise squares. Strictly significant (p < 0.05/141) effects are shown as full symbols; otherwise as empty symbols. LDL = low-density lipoprotein; HDL = high-density lipoprotein (see Table S2).*

Reviewer 5

This study presents a comprehensive analysis on the relationships between leukocyte telomere length (LTL) and 141 complex trait that are closely related to human health.

Mendelian Randomization (MR) and MVMR are used as the main method for the analyses and have revealed several interesting results. My specific comments on the methods and results are as follows:

From a general perspective, we discern three main points that we have addressed in the revised manuscript:

1) Robustness of MR results considering potential bias: To address concerns of sample overlap and pleiotropy, we now implemented the MR-APSS method as suggested. We also replicated our analyses using independent LTL summary statistics, confirming the consistency of our findings.

2) Perform MR for sex-stratified analysis: As we restructure the manuscript, the sex-stratified analyses were removed from the manuscript.

3) Clarity: We have enhanced the clarity of specific sections of the manuscript that required further explanation or detail.

1. In the mediation analysis section, it appears that the authors employed MVMR analysis by using Trait 1 and leukocyte telomere length (LTL) as exposures, while considering Trait 2 as the outcome. This analysis aimed to assess both the direct effect of Trait 1 on Trait 2 and the potential mediated effect of LTL on Trait 2. However, it raises the question of how the mediated effect can be ensured to be independent of other confounding factors, such as Trait 3. Is it possible to conduct MVMR analysis by incorporating additional potential confounders to address this concern?

In the methods section, we have now clarified that the mediator is also instrumented. This means we address bias from the mediator to the outcome in the same way we handle bias from exposure to outcome in typical MR analyses (lines 428-433):

“*IVs for mediation analyses were selected from summary statistics through a two-phase clumping process (see Bidirectional univariable Mendelian randomization) in which harmonized exposure IVs (i.e., trait IVs) were first independently clumped. In the second phase, exposure and mediator IVs (i.e., LTL IVs) were clumped together, prioritizing the former over the latter (i.e., retaining exposure IVs over mediator IVs).* ***Providing MR assumptions hold, by instrumenting both the exposure and mediator we also reduce confounding bias between mediator and outcome.***”

This information has also been added in the Figure 1 caption:

“***...Note that for mediation analyses, both exposure and mediator are instrumented.***”

It is possible to conduct MVMR with multiple exposures, as has been done to disentangle specific examples (e.g., Figure 4b). We now added an MVMR analysis with blood counts and clonal haematopoiesis of indeterminate potential (CHIP) as covariates, which we now present as follows (lines 96-99):

“***MVMR analysis with blood counts (with significant association with LTL), MCH, CHIP, and LTL as exposures against LTL-impacted traits also did not reveal significant effect changes in effect sizes, confirming that neither CHIP nor other hematological parameters biased our results (see Table S3).***”

The incorporation of additional potential confounders into the analysis without preliminary evidence or theoretical rationale can increase bias from the selection of additional confounders (see PMID: 23162385). Moreover, in MVMR analyses, the addition of additional covariates increases the number of genetic instruments. This increases risk for weak instrumental bias and decreases the conditional F-statistic, which can bias the estimated effect of the focal variable towards null due to regression dilution bias.

2. In the mediation analysis section, the authors state, "We tested the mediating role of leukocyte telomere length (LTL) for the relation between 18 non-hematological LTL-modulating traits and lifespan,……". Based on this statement, it can be interpreted that both LTL and the 18 LTL-modulating traits are indeed used as exposures to estimate their individual effects on lifespan. However, considering the results you have presented, it seems the authors used each of the 18 LTL-modulating traits is incorporated individually in a two-exposure MVMR analysis to assess its specific impact on lifespan. To avoid confusion, it could be better the authors make it more clear how MVMR is used in your mediation analysis.

To avoid confusion, we revised the manuscript as follows (lines 423-426):

“*Excluding hematological traits due to potential confounding,* ***we used a two-exposure multivariable MR (MVMR) framework to individually assess the mediating role of LTL across 18 LTL-affecting traits*** *(p < 0.05/141 = 3.5e−4) on ifespan, proxied through parental lifespan [4]. We further examined the global mediatory role of LTL between each of these 18 LTL-affecting traits and 17 traits causally impacted by LTL (p < 0.05/141 = 3.5e−4).*”

3. Given the availability of individual-level data for multiple traits, why the authors do not consider an MR analysis using individual-level data for the sex-stratified analysis, rather than solely relying on association analysis.

Because of the lack of sex-specific summary statistics, the original manuscript did not contain sex-specific MR results. While it is true that availability of individual-level data would allow us to calculate sex-specific MR effects, we have decided to refocus the manuscript and omit any sex-specific results, leaving this for future studies. Sex-stratified analyses may reveal sex-specific effects at the cost of increased multiple testing burden, reduced statistical power (both due to reduced sample size and detecting interaction effects).

4. In the MR analyses, the authors have indeed considered sensitivity analysis using methods like MR-Egger and weighted-mode. However, it is important to acknowledge that these methods are primarily designed to address uncorrelated pleiotropy. Other confounding factors, such as correlated pleiotropy and population stratification, and sample overlap, can also contribute to false positives in MR analysis. It would be valuable if the authors consider sensitivity analysis using methods specifically designed to handle these types of confounding factors, such as CAUSE (PMID: 32451458) and MR-APSS (PMID: 35787050). Additionally, the use of negative controls can be helpful in examining confounding factors.

The revised manuscript now includes MR-APSS among our sensitivity analyses to address correlated/uncorrelated pleiotropy, as well as sample structure simultaneously. Estimates from MR-APSS are in agreement with those obtained from other methods, strengthening the robustness of our findings.

The methods section has been updated as follows (lines 415-421):

“***While sample overlap in two-sample MR may bias results toward observational effects, no overlap may also lead to ’winner’s curse’ bias [27, 28]. Weak instruments can further exacerbate these biases. Although our extensive simulations have demonstrated that these issues lead to mild biases [28], we used MR-APSS [29] (default parameters and LD scores from 1000 Genomes Data), which addresses both sample overlap and pleiotropy. Finally, we replicated the MR analyses using LTL summary statistics generated based on an independent sample (N = 62, 271) from the biobank of Vanderbilt University Medical Center (BioVU) and Marshfield Clinic’s personalized medicine research project (PMRP) [5, 30, 31].*”**

And outcomes of the analysis are described in the added “***Sensitivity analyses***” section (lines 70-83):

“***To ensure the robustness and reliability of the results, we gauged the reliability of inverse-variance weighted (IVW) significant associations through several approaches (Figure 1). First, we estimated robustness towards MR assumption violation. We applied four additional MR methods implemented in the TwoSampleMR package (MR Egger, simple mode, weighted median, and weighted mode), as well as MR-PRESSO (Figures S3-S4). To mitigate pleiotropy bias, we further implemented a custom approach based on Steiger filtering that requires instrumental variables (IVs) to have a stronger association with the exposure (i.e., LTL) than with any of the other 152 traits analyzed through MR (see Methods; Figure S5). To determine the impact of sample structure, and more notably sample overlap, we ran MR-APSS (which also accounts for pleiotropy) and replicated our IVW results using independent LTL summary statistics from BioVU/PMRP [5]. Estimates were globally consistent across all methods (Figures S3-S4). Regarding replication in BioVU/PMRP, the smaller sample size (N = 62, 271) resulted in larger confidence intervals (CI), yet the correlation between effect sizes remained high (ρ_LTL→T_ = 0.930; ρ_T→LTL_ = 0.874). Specifically, 9 LTL on trait and 5 trait on LTL effects strictly replicated (p < 3.5e−4), while 17 and 12 reached nominal significance, respectively. Only the effect of white blood cell (WBC) counts on LTL had a significantly different effect size (p_diff._ < 0.05/47 = 0.001).***”

Inspired by Sanderson *et al*. 2021 (PMID: 33570130), we furthermore included a negative control that assesses the impact of LTL on sex, for which we do not expect any significant causal effect:

“***Third, we performed a negative control. As sex of an individual is determined prior to adult LTL, we should not observe any causal link from LTL to sex [50]. As expected, we did not find a significant causal IVW MR effect of LTL on sex (p = 0.656).*** *To conclude, the broad range of sensitivity analyses we performed showed that our results are globally robust to assumption violation and confounding, allowing their biological interpretation.*”

5. The authors have revealed several findings in their analysis, for example MR analysis have identified 23 traits that modulate LTL. I think it could be beneficial to consider replication studies using independent datasets to validate these findings.

The bidirectional MR analyses have now been replicated with independent LTL data as was presented in the previous point. Overall, we obtained highly concordant results.

6. I find the presentation of the results in Figure 2 to be somewhat cluttered and challenging to discern relevant and significant information.

We appreciate the reviewer’s concern. However, consolidating all the data into one figure also enhances its value, allowing readers to quickly access all necessary information in one place. To make it easier to discern relevant information, in the revised figure, we colored the y-labels in blue if there was a trait to LTL MR effect, in red for LTL to trait MR effect, and in purple for a bidirectional MR effect. The figure caption was adapted accordingly.

***Figure 2. Observational and causal associations between traits and LTL.*** *Estimates (x-axis) with 95% confidence intervals (CI) for traits (y-axis) with at least one strictly significant (p < 0.05/141 = 3.5e−4) association with LTL across the observational correlation (linear regression; β; black) and inverse-variance weighted (IVW) Mendelian randomization (MR) estimates of LTL on trait (α; red) and trait on LTL (α; blue) are shown. Strictly significant effects are shown as full circles; otherwise as empty circles. Traits are colored according to their MR effects, with red, blue, and purple indicating a significant LTL to trait, trait to LTL, and bidirectional effect. For diseases (*), one standard deviation (SD) change in LTL corresponds to one log(OR) change, implying a scale of SDLTL/ log(OR) for the effects of diseases on LTL, and log(OR)/SDLTL for the effect of LTL on the disease, so that observational effects and MR effects are not directly comparable (see Table S2).*

**Refereces**

[1] Geraldine Aubert, Gabriela M. Baerlocher, Irma Vulto, Steven S. Poon, and Peter M. Lansdorp. Collapse of Telomere Homeostasis in Hematopoietic Cells Caused by Heterozygous Mutations in Telomerase Genes. *PLos Genet*., 8(5):e1002696, May 2012. ISSN 1553-7404. doi: 10.1371/journal.pgen.1002696.

[2] Veryan Codd, Qingning Wang, Elias Allara, Crispin Musicha, Stephen Kaptoge, Svetlana Stoma, Tao Jiang, Stephen E. Hamby, Peter S. Braund, Vasiliki Bountziouka, Charley A. Budgeon, Matthew Denniff, Chloe Swinfield, Manolo Papakonstantinou, Shilpi Sheth, Dominika E. Nanus, Sophie C. Warner, Minxian Wang, Amit V. Khera, James Eales, Willem H. Ouwehand, John R. Thompson, Emanuele Di Angelantonio, Angela M. Wood, Adam S. Butterworth, John N. Danesh, Christopher P. Nelson, and Nilesh J. Samani. Polygenic basis and biomedical consequences of telomere length variation. *Nature Genetics*, 53(10):1425–1433, October 2021. ISSN 1546-1718. doi: 10.1038/s41588-021-00944-6.

[3] Adriaan van der Graaf, Maria M. Zorro, Annique Claringbould, Urmo Vosa, Raul Aguirre-Gamboa, Chan Li, Joram Mooiweer, Isis Ricano-Ponce, Zuzanna Borek, Frits Koning, Yvonne Kooy-Winkelaar, Ludvig M. Sollid, Shuo-Wang Qiao, Vinod Kumar, Yang Li, Lude Franke, Sebo Withoff, Cisca Wijmenga, Serena Sanna, Iris Jonkers, and BIOS Consortium. Systematic Prioritization of Candidate Genes in Disease Loci Identifies TRAFD1 as a Master Regulator of IFNγ Signaling in Celiac Disease. *Frontiers in Genetics*, 11:562434, January 2021. ISSN 1664-8021. doi: 10.3389/fgene.2020.562434.

[4] Paul Rhj Timmers, Ninon Mounier, Kristi Lall, Krista Fischer, Zheng Ning, Xiao Feng, Andrew D. Bretherick, David W. Clark, eQTLGen Consortium, Xia Shen, Tonu Esko, Zoltan Kutalik, James F. Wilson, and Peter K. Joshi. Genomics of 1 million parent lifespans implicates novel pathways and common diseases and distinguishes survival chances. *eLife,* January 2019. doi: 10.7554/eLife.39856.

[5] Patrick Allaire, Jing He, John Mayer, Luke Moat, Peter Gerstenberger, Reynor Wilhorn, Sierra Strutz, David S. L. Kim, Chenjie Zeng, Nancy Cox, Jerry W. Shay, Joshua Denny, Lisa Bastarache, and Scott Hebbring. Genetic and clinical determinants of telomere length. *Human Genetics and Genomics Advances*, 4(3), July 2023. ISSN 2666-2477. doi: 10.1016/j.xhgg.2023.100201.

[6] Wenxiu Wang, Ninghao Huang, Zhenhuang Zhuang, Zimin Song, Yueying Li, Xue Dong, Wendi Xiao, Yimin Zhao, Jinzhu Jia, Zhonghua Liu, et al. Identifying Potential Causal Effects of Telomere Length on Health Outcomes: A Phenome-Wide Investigation and Mendelian Randomization Study. *Journals of Gerontology*: Series A, page glad128, May 2023. ISSN 1758-535X. doi: 10.1093/gerona/glad128.

[7] Kathryn Demanelis, Lin Tong, and Brandon L. Pierce. Genetically Increased Telomere Length and Aging-Related Traits in the U.K. Biobank. *Journals of Gerontology*: Series A, 76(1):15–22, January 2021. ISSN 1079-5006. doi: 10.1093/gerona/glz240.

[8] Zhiyou Cai, Liang-Jun Yan, and Anna Ratka. Telomere Shortening and Alzheimer’s Disease. *NeuroMolecular Medicine*, 15(1):25–48, March 2013. ISSN 1559-1174. doi: 10.1007/s12017-012-8207-9.

[9] Kai Gao, Xin Wang, Weihua Yue, and Hao Yu. Exploring the Causal Pathway From Telomere Length to Alzheimer’s Disease: An Update Mendelian Randomization Study. *Frontiers in Psychiatry*, 10:489035, November 2019. ISSN 1664-0640. doi: 10.3389/fpsyt.2019.00843.

[10] Tina Levstek, Eva Kozjek, Vita Dolzan, and Katarina Trebusak Podkrajsek. Telomere Attrition in Neurodegenerative Disorders. *Frontiers in Cellular Neuroscience*, 14:556488, July 2020. ISSN 1662-5102. doi: 10.3389/fncel.2020.00219.

[11] Lana Fani, Saima Hilal, Sanaz Sedaghat, Linda Broer, Silvan Licher, Pascal P. Arp, Joyce B. J. van Meurs, M. Kamran Ikram, and M. Arfan Ikram. Telomere Length and the Risk of Alzheimer’s Disease: *The Rotterdam Study. Journal of Alzheimer’s Disease*, 73(2):707–714, January 2020. ISSN 1387-2877. doi: 10.3233/JAD-190759.

[12] Fernanda Schafer Hackenhaar, Maria Josefsson, Annelie Nordin Adolfsson, Mattias Landfors, Karolina Kauppi, Magnus Hultdin, Rolf Adolfsson, Sofie Degerman, and Sara Pudas. Short leukocyte telomeres predict 25-year Alzheimer’s disease incidence in non-APOE ϵ4-carriers. *Alzheimer’s Research & Therapy*, 13(1):1–13, December 2021. ISSN 1758-9193. doi: 10.1186/s13195-021-00871-y.

[13] Guangping Yu, Leihong Lu, Zaihong Ma, and Shouhai Wu. Genetically Predicted Telomere Length and Its Relationship With Alzheimer’s Disease. *Frontiers in Genetics*, 12:595864, February 2021. ISSN 1664-8021. doi: 10.3389/fgene.2021.595864.

[14] Xu-Fan Wang, Wen-Jing Xu, Fei-Fei Wang, Rui Leng, Xiao-Ke Yang, Hua-Zhi Ling, Yin-Guang Fan, Jin-Hui Tao, Zong-Wen Shuai, Li Zhang, et al. Telomere Length and Development of Systemic Lupus Erythematosus: A Mendelian Randomization Study. *Arthritis & Rheumatology*, 74 (12):1984–1990, December 2022. ISSN 2326-5191. doi: 10.1002/art.42304.

[15] Meiling Liu, Ping Luo, Lihua Liu, Xianping Wei, Xue Bai, Jicui Li, Linlin Wu, and Manyu Luo. Immune-mediated inflammatory diseases and leukocyte telomere length: A Mendelian randomization study. *Frontiers in Genetics*, 14:1129247, April 2023. ISSN 1664-8021. doi: 10.3389/fgene.2023.1129247.

[16] Bangbei Wan, Likui Lu, and Cai Lv. Mendelian randomization study on the causal relationship between leukocyte telomere length and prostate cancer. *PLOS ONE*, 18(6):e0286219, June 2023. ISSN 1932-6203. doi: 10.1371/journal.pone.0286219.

[17] A. Topiwala, B. Taschler, K. P. Ebmeier, S. Smith, H. Zhou, D. F. Levey, V. Codd, N. J. Samani, J. Gelernter, T. E. Nichols, et al. Alcohol consumption and telomere length: Mendelian randomization clarifies alcohol’s effects. *Molecular Psychiatry*, 27(10):4001–4008, October 2022. ISSN 1476-5578. doi: 10.1038/s41380-022-01690-9

[18] Yuliana Astuti, Ardyan Wardhana, Johnathan Watkins, Wahyu Wulaningsih, and For the Pilar Research Network. Cigarette smoking and telomere length: A systematic review of 84 studies and meta-analysis. *Environmental Research*, 158:480, October 2017. doi: 10.1016/j.envres.2017.06.038.

[19] Sehoon Park, Seong Geun Kim, Soojin Lee, Yaerim Kim, Semin Cho, Kwangsoo Kim, Yong Chul Kim, Seung Seok Han, Hajeong Lee, Jung Pyo Lee, et al. Causal linkage of tobacco smoking with ageing: Mendelian randomization analysis towards telomere attrition and sarcopenia. *Journal of Cachexia, Sarcopenia and Muscle*, 14(2):955–963, April 2023. ISSN 2190-5991. doi: 10.1002/jcsm.13174.

[20] Bangbei Wan, Ning Ma, and Cai Lv. Identifying effects of genetic obesity exposure on leukocyte telomere length using Mendelian randomization. *PeerJ*, 11:e15085, March 2023. ISSN 2167-8359. doi: 10.7717/peerj.15085.

[21] Belinda L. Needham, Bilinda Straight, Charles E. Hilton, Charles Owuor Olungah, and Jue Lin. Family socioeconomic status and child telomere length among the Samburu of Kenya. *Social Science & Medicine*, 283:114182, August 2021. ISSN 0277-9536. doi: 10.1016/j.socscimed.2021.114182.

[22] Vikesh Amin, Jason M. Fletcher, Zhongxuan Sun, and Qiongshi Lu. Higher educational attainment is associated with longer telomeres in midlife: Evidence from sibling comparisons in the UK Biobank. *SSM - Population Health*, 17:101018, March 2022. ISSN 2352-8273. doi: 10.1016/j.ssmph.2021.101018.

[23] Pedro A. Pousa, Raquel M. Souza, Paulo Henrique M. Melo, Bernardo H. M. Correa, Tamires S. C. Mendonca, Ana Cristina Simoes-e. Silva, and D ´ebora M. Miranda. Telomere Shortening and Psychiatric Disorders: A Systematic Review. *Cells*, 10(6):1423, June 2021. ISSN 2073- 4409. doi: 10.3390/cells10061423.

[24] Einat Rauchbach, Haim Zeigerman, Diana Abu-Halaka, and Oren Tirosh. Cholesterol Induces Oxidative Stress, Mitochondrial Damage and Death in Hepatic Stellate Cells to Mitigate Liver Fibrosis in Mice Model of NASH. *Antioxidants*, 11(3):536, March 2022. ISSN 2076-3921. doi: 10.3390/antiox11030536.

[25] Thomas von Zglinicki. Oxidative stress shortens telomeres. *Trends in Biochemical Sciences*, 27(7):339–344, July 2002. ISSN 0968-0004. doi: 10.1016/S0968-0004(02)02110-2.

[26] Gehua Zhu, Jiamin Xu, Guanghua Guo, and Feng Zhu. Association between Lipids, Apolipoproteins and Telomere Length: A Mendelian Randomization Study. *Nutrients*, 15(21):4497, October 2023. ISSN 2072-6643. doi: 10.3390/nu15214497.

[27] Stephen Burgess, Neil M. Davies, and Simon G. Thompson. Bias due to participant overlap in two-sample Mendelian randomization. *Genetic Epidemiology*, 40(7):597–608, November 2016. ISSN 0741-0395. doi: 10.1002/gepi.21998.

[28] Ninon Mounier and Zoltan Kutalik. Bias correction for inverse variance weighting Mendelian randomization. *Genetic Epidemiology*, 47(4):314–331, June 2023. ISSN 0741-0395. doi: 10.1002/gepi.22522.

[29] Xianghong Hu, Jia Zhao, Zhixiang Lin, Yang Wang, Heng Peng, Hongyu Zhao, Xiang Wan, and Can Yang. Mendelian randomization for causal inference accounting for pleiotropy and sample structure using genome-wide summary statistics. *Proceedings of the National Academy of Sciences*, 119(28):e2106858119, July 2022. doi: 10.1073/pnas.2106858119.

[30] Catherine A. McCarty, Russell A. Wilke, Philip F. Giampietro, Steve D. Wesbrook, and Michael D. Caldwell. Marshfield Clinic Personalized Medicine Research Project (PMRP): design, methods and recruitment for a large population-based biobank. *Personalized Medicine*, November 2005. doi: 10.1517/17410541.2.1.49.

[31] Jill Pulley, Ellen Clayton, Gordon R. Bernard, Dan M. Roden, and Daniel R. Masys. Principles of Human Subjects Protections Applied in an Opt-Out, De-identified Biobank. *Clinical and Translational Science*, 3(1):42–48, February 2010. ISSN 1752-8054. doi: 10.1111/j.1752-8062.2010.00175.x.

[32] Gibran Hemani, Jie Zheng, Benjamin Elsworth, Kaitlin H. Wade, Valeriia Haberland, Denis Baird, Charles Laurin, Stephen Burgess, Jack Bowden, Ryan Langdon, Vanessa Y. Tan, James Yarmolinsky, Hashem A. Shihab, Nicholas J. Timpson, David M. Evans, Caroline Relton, Richard M. Martin,

George Davey Smith, Tom R. Gaunt, and Philip C. Haycock. The MR-Base platform supports systematic causal inference across the human phenome. *eLife*, May 2018. doi: 10.7554/eLife.34408.

[33] Christopher C. Chang, Carson C. Chow, Laurent Cam Tellier, Shashaank Vattikuti, Shaun M. Purcell, and James J. Lee. Second-generation PLINK: rising to the challenge of larger and richer datasets. *GigaScience*, 4(1):13742–015, December 2015. ISSN 2047-217X. doi: 10.1186/s13742-015-0047-8.

[34] Felix Molder, Kim Philipp Jablonski, Brice Letcher, Michael B. Hall, Christopher H. Tomkins-Tinch, Vanessa Sochat, Jan Forster, Soohyun Lee, Sven O. Twardziok, Alexander Kanitz, Andreas Wilm, Manuel Holtgrewe, Sven Rahmann, Sven Nahnsen, and Johannes Koster. Sustainable data analysis with Snakemake. *F1000Research*, 10(33):33, April 2021. doi: 10.12688/f1000research.29032.2.

[35] Stephen Burgess and Jeremy A. Labrecque. Mendelian randomization with a binary exposure variable: interpretation and presentation of causal estimates. *European Journal of Epidemiology*, 33(10):947–952, October 2018. ISSN 1573-7284. doi: 10.1007/s10654-018-0424-6.

[36] Tetsushi Nakao, Alexander G. Bick, Margaret A. Taub, Seyedeh M. Zekavat, Md M. Uddin, Abhishek Niroula, Cara L. Carty, John Lane, Michael C. Honigberg, Joshua S. Weinstock, et al. Mendelian randomization supports bidirectional causality between telomere length and clonal hematopoiesis of indeterminate potential. *Science Advances*, 8(14), April 2022. ISSN 2375-2548. doi: 10.1126/sciadv.abl6579.

[37] Emily A. DeBoy, Michael G. Tassia, Kristen E. Schratz, Stephanie M. Yan, Zoe L. Cosner, Emily J. McNally, Dustin L. Gable, Zhimin Xiang, David B. Lombard, Emmanuel S. Antonarakis, Christopher D. Gocke, Rajiv C. McCoy, and Mary Armanios. Familial Clonal Hematopoiesis in a Long Telomere Syndrome. *New England Journal of Medicine*, 388(26):2422–2433, May 2023. ISSN 1533-4406. doi:

10.1056/NEJMoa2300503.

[38] Michael D. Kessler, Amy Damask, Sean O’Keeffe, Nilanjana Banerjee, Dadong Li, Kyoko Watanabe, Anthony Marketta, Michael Van Meter, Stefan Semrau, Julie Horowitz, et al. Common and rare variant associations with clonal haematopoiesis phenotypes. *Nature*, 612(7939):301–309, December 2022. ISSN 1476-4687. doi: 10.1038/s41586-022-05448-9.

[39] Masood A. Shammas. Telomeres, lifestyle, cancer, and aging. *Current Opinion in Clinical Nutrition & Metabolic Care*, 14(1):28, January 2011. ISSN 1363-1950. doi: 10.1097/MCO.0b013e32834121b1.

[40] Samuel R. Chamberlain, Jonathan Cavanagh, Peter de Boer, Valeria Mondelli, Declan N. C. Jones, Wayne C. Drevets, Philip J. Cowen, Neil A. Harrison, Linda Pointon, Carmine M. Pariante, et al. Treatment-resistant depression and peripheral C-reactive protein. *British Journal of Psychiatry*, 214(1):11–19, January 2019. ISSN 0007-1250. doi: 10.1192/bjp.2018.66.

[41] Pedro A. Pousa, Raquel M. Souza, Paulo Henrique M. Melo, Bernardo H. M. Correa, Tamires S. C. Mendonc¸a, Ana Cristina Simoes-e. Silva, and Debora M. Miranda. Telomere Shortening and Psychiatric Disorders: A Systematic Review. *Cells*, 10(6):1423, June 2021. ISSN 2073-4409. doi: 10.3390/cells10061423.

[42] Zhengtao Lv, Jiarui Cui, and Jiaming Zhang. Associations between serum urate and telomere length and inflammation markers: Evidence from UK Biobank cohort. *Frontiers in Immunology*, 13:1065739, December 2022. ISSN 1664-3224. doi: 10.3389/fimmu.2022.1065739.

[43] Masafumi Kurajoh, Shinya Fukumoto, Shio Yoshida, Seigo Akari, Takayo Murase, Takashi Nakamura, Haruka Ishii, Hisako Yoshida, Yuki Nagata, Tomoaki Morioka, et al. Uric acid shown to contribute to increased oxidative stress level independent of xanthine oxidoreductase activity in MedCity21 health examination registry. *Scientific Reports*, 11(7378):1–9, April 2021. ISSN 2045-2322. doi: 10.1038/s41598-021-86962-0.

[44] Vasimahmed Lala, Muhammad Zubair, and David A. Minter. Liver Function Tests. In StatPearls [Internet]. *StatPearls* Publishing, July 2023. URL https://www.ncbi.nlm.nih.gov/books/NBK482489.

[45] Mrinal M. Patnaik, Patrick S. Kamath, and Douglas A. Simonetto. Hepatic manifestations of telomere biology disorders. *Journal of Hepatology*, 69(3):736–743, September 2018. ISSN 0168-8278. doi: 10.1016/j.jhep.2018.05.006.

[46] Kathryn Demanelis, Farzana Jasmine, Lin S. Chen, Meytal Chernoff, Lin Tong, Dayana Delgado, Chenan Zhang, Justin Shinkle, Mekala Sabarinathan, Hannah Lin, et al. Determinants of telomere length across human tissues. *Science* (New York, N.Y.), 369(6509):eaaz6876., September 2020. ISSN 1095-9203. doi: 10.1126/science.aaz6876

[47] Liza Darrous, Ninon Mounier, and Zoltan Kutalik. Simultaneous estimation of bi-directional causal effects and heritable confounding from GWAS summary statistics. *Nature Communications*, 12(7274):1–15, December 2021. ISSN 2041-1723. doi: 10.1038/s41467-021-26970-w.

[48] Thomas Tsiampalis and Demosthenes B. Panagiotakos. Missing-data analysis: socio demographic, clinical and lifestyle determinants of low response rate on self- reported psychological and nutrition related multi- item instruments in the context of the ATTICA epidemiological study. *BMC Medical Research Methodology*, 20(1):1–13, December 2020. ISSN 1471-2288. doi: 10.1186/s12874-020-01038-3.

[49] Aladdin H. Shadyab, Margery L. S. Gass, Marcia L. Stefanick, Molly E. Waring, Caroline A. Macera, Linda C. Gallo, Richard A. Shaffer, Sonia Jain, and Andrea Z. LaCroix. Maternal Age at Childbirth and Parity as Predictors of Longevity Among Women in the United States: The Women’s Health Initiative. *American Journal of Public Health*, December 2016. URL https://ajph.aphapublications.org/doi/abs/10.2105/AJPH.2016.303503?journalCode=ajph.

[50] Eleanor Sanderson, Tom G. Richardson, Gibran Hemani, and George Davey Smith. The use of negative control outcomes in Mendelian randomization to detect potential population stratification. *International Journal of Epidemiology*, 50(4):1350–1361, August 2021. ISSN 0300-5771. doi: 10.1093/ije/dyaa288.

**2^nd^ round**

**Reviewer 1**

All my comments have been addressed.

**Reviewer 2**

I would like to express my appreciation to the authors for their diligent revisions and efforts in addressing the concerns raised in my previous review. The revised manuscript reflects a significant improvement and serves as an exemplary model for a Mendelian randomization (MR) study. I have a few minor comments for further consideration:

1. MR Egger Intercepts: I am grateful for the inclusion of the MR Egger intercept in supplementary Table 2. However, upon reviewing Table ST2, I observed that analyses such as age_end_education, age_first_birth (AFB), and age_last_birth (ALB) in the reverse MR exhibited significant non-zero MR Egger intercepts (P < 0.05), indicating potential directional pleiotropy. This unbalanced pleiotropy could introduce bias to the derived causal effect estimate and warrants discussion as a limitation at least.

2. Validation in BioVU/PMRP: While IVW results are presented for validation, I suggest including the results of other sensitivity analyses such as MR Egger, Median, Mode, etc.

3. Supplementary Table ST5: It would be beneficial to include a heterogeneity test or "Pdiff" for a quantitative comparison between the current and previous studies. Additionally, transforming odds ratios from previous publications into the beta scale would facilitate a head-to-head comparison.

4. Weak Instrument Bias in MVMR: The conditional F-statistics displayed in Table S3 mostly fall below 10, indicating potential susceptibility to weak instrument bias and lack of independence as instruments. This should be acknowledged and discussed as a limitation (Sanderson et al 2021; https://doi.org/10.1002/sim.9133 ).

5. HLA Region Exclusion: Please provide a reference supporting the statement "This practice aligns with common strategies in the field aimed at minimizing bias from pleiotropy and ensuring clearer, more interpretable results."

6. In addition to these points, I found the relationship between AFB/ALB and LTL to be an intriguing finding. It would be valuable if the authors could discuss the recent paper on the effects of pregnancy and its progression on human biological aging (Pham et al. 2024; https://doi.org/10.1016/j.cmet.2024.02.016). However, feel free to disregard this suggestion if it is deemed irrelevant.

Overall, I commend the authors for their meticulous work and look forward to seeing the final version of the manuscript.

**Authors’ response**

Reviewer 1

no further comments

We thank Reviewer #1 for their positive feedback. We are glad that our revisions met their expectations, and we appreciate the time they dedicated to reviewing our work.

Reviewer 2

I would like to express my appreciation to the authors for their diligent revisions and efforts in addressing the concerns raised in my previous review. The revised manuscript reflects a significant improvement and serves as an exemplary model for a Mendelian randomization (MR) study. I have a few minor comments for further consideration:

We are grateful to Reviewer #2 for their detailed and insightful feedback, which has significantly improved our manuscript. In the revised manuscript, we discuss the remaining possible biases and added the requested additional metrics in the supplementary tables.

1. MR Egger Intercepts: I am grateful for the inclusion of the MR Egger intercept in supplementary Table 2. However, upon reviewing Table ST2, I observed that analyses such as age_end_education, age_first_birth (AFB), and age_last_birth (ALB) in the reverse MR exhibited significant non-zero MR Egger intercepts (P < 0.05), indicating potential directional pleiotropy. This unbalanced pleiotropy could introduce bias to the derived causal effect estimate and warrants discussion as a limitation at least.

Indeed, significant MR-Egger intercepts may indicate directional pleiotropy. However, testing whether the intercept is significant also requires correction for multiple testing. While five intercepts were nominally significant, none of them survived multiple testing correction, hence we have no solid evidence for directional pleiotropy. To be fully transparent, we explicitly state when there is a nominal trend for directional pleiotropy, as is the case or age at first and last live birth:

“*Both traits also causally influenced LTL (AFB: α_T→LTL_ = 0.167; p = 1.2e−5; ALB: α_T→LTL_ = 0.272; p = 6.1e−6), suggesting that timing of female reproductive events could modulate LTL.* ***While these effects showed nominally significant MR-Egger intercepts, potentially indicating directional pleiotropy, the latter did not survive multiple testing corrections (Additional file 2: Table S2).***”

2. Validation in BioVU/PMRP: While IVW results are presented for validation, I suggest including the results of other sensitivity analyses such as MR Egger, Median, Mode, etc.

We have now included the results of MR Egger, simple mode, weighted median, and weighted mode with the LTL validation data in Supplementary Table 2 (Table S2). Since it was only a replication analysis, we decided not to include other MR methods.

3. Supplementary Table ST5: It would be beneficial to include a heterogeneity test or "Pdiff" for a quantitative comparison between the current and previous studies. Additionally, transforming odds ratios from previous publications into the beta scale would facilitate a head-to-head comparison.

We have converted the odds ratio estimates to the beta scale and performed a two-sided t-test to compute the "P_diff._" which is now included in Supplementary Table 5 (Table S5). Only the effects of LTL on ischemic heart disease and kidney cancer showed nominally (p < 0.05) significant differences, although the direction of the estimates was consistent.

4. Weak Instrument Bias in MVMR: The conditional F-statistics displayed in Table S3 mostly fall below 10, indicating potential susceptibility to weak instrument bias and lack of independence as instruments. This should be acknowledged and discussed as a limitation (Sanderson et al. 2021; https://doi.org/10.1002/sim.9133).

The limitations section has now been revised accordingly to address this concern:

“*Fourth, MR presents with inherent limitations, notably susceptibility to horizontal pleiotropy violations, especially given the considerable heterogeneity across our IVs.* ***While MVMR analyses can mitigate biases introduced by pleiotropy and elucidate direct causal effects, these analyses are also more likely to be subject to weak instrument bias, which is indicated by several conditional F-statistics falling below 10 (Additional file 2: Table S3) [1]****.*”

5. HLA Region Exclusion: Please provide a reference supporting the statement "This practice aligns with common strategies in the field aimed at minimizing bias from pleiotropy and ensuring clearer, more interpretable results."

In the manuscript we state: “*Due to the complex long-range linkage disequilibrium (LD) structure of the HLA locus, SNPs mapping to that region (chr6:25'000'000–37'000'000; GRCh37/hg19) were also excluded from our IVs [2]*”.

As previously stated, although removing HLA IV may reduce the power to detect HLA-mediated associations, we believe that it enhances the reliability of our findings and may in a worst-case scenario only lead to false negatives for HLA-mediated associations. While indeed not explicitly stated, this strategy is used in several papers. For example https://doi.org/10.1038/s41588-020-0682-6 “Due to the complex LD structure of SNPs within the human major histocompatibility complex (MHC) region, we removed SNPs and proteins encoded by genes within the MHC region (chr6: from 26 Mb to 34 Mb”), https://doi.org/10.1016/j.xcrm.2022.100805 “Although putative causal pathways associated with the HLA region are intriguing, mapping of the SNPs within the HLA region to genes is

challenging because of the extensive LD across the region… HLA-region SNPs were conservatively removed from MR analyses by excluding the short-arm of chromosome 6”, and https://doi.org/10.1073/pnas.2106858119 (MHC-region SNP removal directly built in the method).

6. In addition to these points, I found the relationship between AFB/ALB and LTL to be an intriguing finding. It would be valuable if the authors could discuss the recent paper on the effects of pregnancy and its progression on human biological aging (Pham et al. 2024; https://doi.org/10.1016/j.cmet.2024.02.016). However, feel free to disregard this suggestion if it is deemed irrelevant.

We agree that this reference is relevant and have already discussed it in the previous version of the manuscript:

“*An alternative explanation is that LTL shortening is driven by the stress imposed by such events on the body, aligning with literature that posits pregnancy as a significant accelerator of biological aging, measured per methylation [3].*”

**References:**

[1] Sanderson E, Spiller W, Bowden J. Testing and correcting for weak and pleiotropic instruments in two-sample multivariable Mendelian randomization. Statistics in Medicine. 2021 Nov;40(25):5434-52.

[2] van der Graaf A, Zorro MM, Claringbould A, Vosa U, Aguirre-Gamboa R, Li C, et al. Systematic Prioritization of Candidate Genes in Disease Loci Identifies TRAFD1 as a Master Regulator of IFNγ Signaling in Celiac Disease. Frontiers in Genetics. 2021 Jan;11:562434.

[3] Pham H, Thompson-Felix T, Czamara D, Rasmussen JM, Lombroso A, Entringer S, et al. The effects of pregnancy, its progression, and its cessation on human (maternal) biological aging. Cell Metabolism. 2024 Mar.
